# Genomic and phenotypic analyses of six offspring of a genome-edited hornless bull

**DOI:** 10.1038/s41587-019-0266-0

**Published:** 2019-10-07

**Authors:** Amy E. Young, Tamer A. Mansour, Bret R. McNabb, Joseph R. Owen, Josephine F. Trott, C. Titus Brown, Alison L. Van Eenennaam

**Affiliations:** 1 Department of Animal Science, University of California, Davis, CA USA; 2 Department of Clinical Pathology, School of Medicine, University of Mansoura, Mansoura, Egypt; 3 Department of Population Health and Reproduction, School of Veterinary Medicine, University of California, Davis, CA USA

**Keywords:** Animal biotechnology, Agricultural genetics, Animal breeding

## Abstract

Genome editing followed by reproductive cloning was previously used to produce two hornless dairy bulls. We crossed one genome-edited dairy bull, homozygous for the dominant P_C_ Celtic *POLLED* allele, with horned cows (pp) and obtained six heterozygous (P_C_p) polled calves. The calves had no horns and were otherwise healthy and phenotypically unremarkable. We conducted whole-genome sequencing of all animals using an Illumina HiSeq4000 to achieve ~20× coverage. Bioinformatics analyses revealed the bull was a compound heterozygote, carrying one naturally occurring P_C_ Celtic *POLLED* allele and an allele containing an additional introgression of the homology-directed repair donor plasmid along with the P_C_ Celtic allele. These alleles segregated in the offspring of this bull, and inheritance of either allele produced polled calves. No other unintended genomic alterations were observed. These data can be used to inform conversations in the scientific community, with regulatory authorities and with the public around ‘intentional genomic alterations’ and future regulatory actions regarding genome-edited animals.

## Abstract (web summary)

A genome-edited hornless compound heterozygous dairy bull transmitted this trait to his offspring.

## Main

In the modern US dairy cattle industry, destruction of horn-producing cells before they grow and attach to the skull (disbudding) is a routine practice to prevent horn growth. Animals that do not have horns do not injure other animals, require less feeding trough space, are less dangerous to handle and transport than horned animals and have fewer aggressive behaviors^1^. Disbudding is an unpleasant process that has important implications for animal welfare, and many stakeholder groups have campaigned for alternative, humane solutions. One option is to select and breed animals that do not have horns, a phenotype referred to as polled.

In 2016, Carlson et al.^2^ reported the introgression of the P_C_ Celtic *POLLED* allele into two male dairy bulls by genome editing using transcription activator-like effector nucleases (TALENs). Bulls RCI001 and RCI002 originated at Recombinetics, Inc., where the researchers genome-edited donor cells from a University of Minnesota crossbred dairy bull and then used reproductive cloning. Whole-genome sequencing (WGS) did not reveal any off-target alterations^2^, and both bulls reached maturity without developing horns. These genome-edited polled bulls were transferred to the University of California (UC), Davis and generated widespread interest. However, further work needs to be done in characterizing these animals if genome editing is to seamlessly integrate into livestock genetic improvement programs.

Edits will likely need to be introduced into multiple elite founder animals to prevent genetic bottlenecks^3^. Perhaps as importantly, appropriate regulatory frameworks that are risk- and evidence-based, proportionate and globally harmonized will be essential to allow research to occur, and to foster the development of useful applications^4^. Others have reported on WGS of trios of genome-edited (CRISPR/Cas9) knockout livestock produced through cytoplasmic injection (CPI) of guide RNA (gRNA) and Cas9 into one-cell-stage zygotes. Genome-edited sheep were compared to their parents^5^ and genome-edited goats were compared to their offspring^6^, and both trio-based studies concluded that de novo mutation rates were comparable to those observed in nonedited trios. A third study used an unbiased WGS on two genome-edited calves produced by a targeted gene knockout of beta-lactoglobulin using CPI of a homology-directed repair (HDR) donor plasmid and TALENs into early zygotes^7^. These calves were free of any TALEN-mediated off-target mutations or donor plasmid integration events.

To provide data to guide emerging regulatory frameworks and benefit future applications of genome editing in livestock, we set up a breeding experiment to investigate whether the *POLLED* genome edit was faithfully passed to offspring and whether there were any unique phenotypic or genotypic changes in those offspring. The calves produced as part of the current study are, to our knowledge, the first reported offspring of a genome-edited bull. These data will help inform regulatory agencies as they formulate processes to regulate genome-edited livestock. Appropriate regulation is of pivotal importance if this technology is to have a role in commercial livestock production, especially in light of the 2017 United States Food and Drug Administration’s Draft Guidance for Industry no. 187, entitled ‘Regulation of Intentionally Altered Genomic DNA in Animals’^8^, which judges intentional DNA alterations as new animal drugs.

## Results

### Breeding of polled calves

**Fig. 1:**
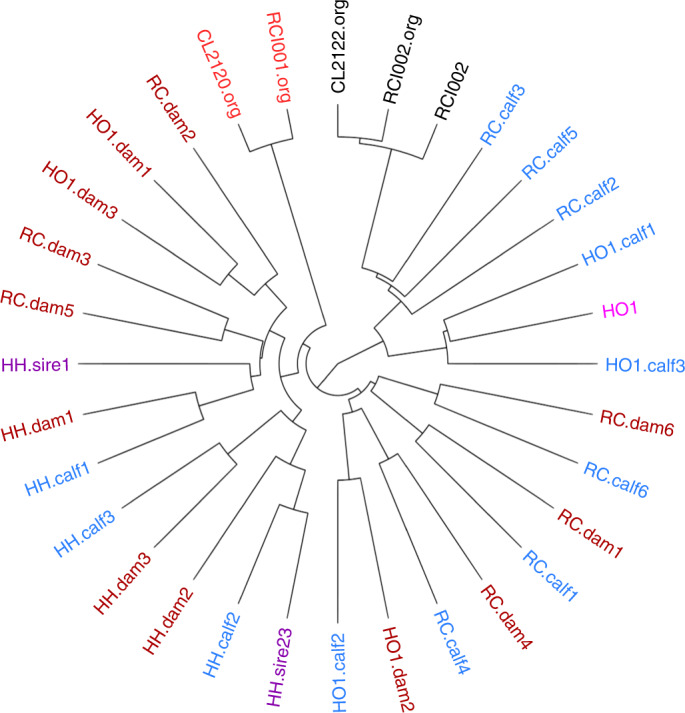
Dendrogram of the phylogenetic relationship (IBS distance) among the sequences analyzed in this study. Less similar sequences have clade branch points closer to the center of the circle. Genome-edited polled bull (RCI002, black) and progenitor cell line (CL2122.org, black); Horned Hereford bulls (purple); Holstein bull (pink); Horned Hereford cows (brown); calves (blue); unrelated genome-edited bull^2^ (RCI001.org, red) and progenitor cell line (CL2120.org, red). The genome-edited polled bull sequence is represented twice; once (RCI002) from sequencing performed as part of this study and once (RCI002.org) as the original sequence reported by Carlson et al.^2^.

**Fig. 2:**
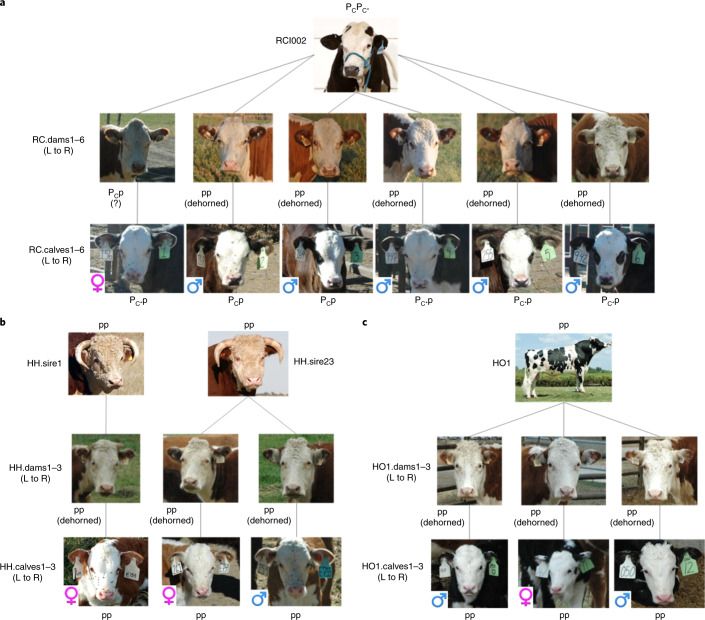
Offspring of the genome-edited polled bull and controls. Shown are the six offspring and six contemporary controls at <3 months of age (before any horn development) and their parents. **a**, Study group GH.H.: the genome-edited polled bull (RCI002) was bred to Horned Hereford cows (RC.dams1–6) and produced six polled offspring (RC.calves1–6). **b**, Study group H.H.: Horned Hereford bulls (HH.sire1 and HH.sire23) bred to Horned Hereford cows (HH.dams1–3) by artificial insemination or natural service produced three horned offspring (HH.calves1–3). **c**, Study group Ho.H.: the Horned Holstein sire (HO1) of the genome-edited polled bull in **a** was bred to Horned Hereford cows (HO1.dams1–3) by artificial insemination and produced three horned offspring (HO1.calves1–3). P_C_ designates the Celtic *POLLED* allele (dominant), P_C_* designates the additional introgression of the HDR donor plasmid along with the P_C_ Celtic allele and p designates the wild type *HORNED* allele (recessive). Offspring are labeled as male or female by blue and pink symbols, respectively. All pictures are of the actual animals, with the exception of the two Horned Hereford bulls, for which the images are representative.

Semen from a genome-edited polled bull (RCI002)^2^ was collected, cryopreserved and used to artificially inseminate ten estrus-synchronized Horned Hereford cows. This bull originated from the University of Minnesota dairy crossbreeding program and is known to be 62.5% Holstein, 25% Montbelliarde and 12.5% Jersey. Six pregnancies resulted, with one female and five male calves born in September 2017. This pregnancy rate of 60% is comparable to those reported under similar estrus-synchronization and artificial insemination protocols^9^. Contemporary controls consisted of purebred Horned Hereford calves (two females and one male born in September 2017). Horned Hereford cows were also bred to the Holstein sire (HO1) of RCI002 by artificial insemination and three calves (one female, two males) were born in December 2017. Figure 1 shows a dendrogram of the identity by state (IBS) distance among the DNA sequences from the 28 cattle (pictured in Fig. 2) involved in this study along with the original sequences from Carlson et al.^2^. Genetic testing verified the parentage of each calf (Methods).

Sequencing data from the same individual performed at different sequencing laboratories (that is, RCI002 and RCI002.org) differed more than the sequences of an edited animal and its unedited progenitor cell line sequenced at the same time and location (for example, CL2122.org and RCI002.org) (Fig. 1). In some cases, the Horned Hereford dams were closely related and cluster together. For example, HO1.dam1 and HO1.dam3 (upper left) are full siblings, and RC.dam2, who groups closely with them, is their half-sibling based on pedigree records.

### Assessment of calf health

The calves were born without incident, with the exception of one Holstein (HO1) × Hereford control calf that was breech and required veterinary intervention at birth. A comprehensive veterinary physical examination was performed on all of the calves at approximately one week of age, including palpation for the presence of horn buds. Horn buds were not present in calves from the genome-edited sire, but were present in Hereford control calves and Holstein × Hereford calves (Fig. 2). All routine physical parameters were within normal limits and comparable between the offspring of the genome-edited polled bulls and control calves. All bull calves had two descended testicles, with the exception of one of the offspring from the genome-edited polled bull (RC.calf6) that had one descended testicle and one cryptorchid testicle external to the inguinal ring, above the neck of the scrotum. Complete blood counts and blood chemistry analyses were performed, with results comparable across all groups of calves.

Additional veterinary physical exams, evaluating the same metrics, were performed at approximately 8 and 12 months of age. All calves were healthy and all parameters were within normal limits. In addition, bull calves in the genome-edited offspring and control offspring groups underwent breeding soundness examinations at 15 months of age, following the standards set out by the Society of Theriogenology^10^. Four bulls from the genome-edited offspring group passed and were classified as satisfactory potential breeders, while one bull (RC.calf6) was unsatisfactory due to an undescended (cryptorchid) testicle. All control bulls were deemed satisfactory potential breeders. No calves in any group had any significant health events during the study timeframe. At the completion of this study, the bull RCI002 and his five male offspring were euthanized and incinerated as their intentional genome edits were unapproved animal drugs^8^, and therefore could not be marketed to enter the food supply.

### Assessment of *POLLED* genotype

**Fig. 3:**
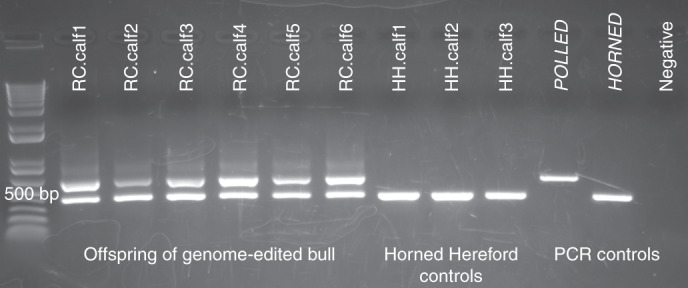
PCR results for the genotypes of the offspring of the genome-edited bull (RC.calves1–6) and three of the contemporary Horned Hereford controls (HH.calves1–3). Homozygous polled (Polled, 591 bp), homozygous horned (Horned, 389 bp) and negative PCR controls are shown at the right. The offspring of the genome-edited polled bull are heterozygous *POLLED* and the Horned Hereford controls are homozygous *HORNED*. This PCR was carried out for the 28 animals sequenced in this study.

Blood samples were collected, DNA extracted and PCR performed to test for *POLLED* and *HORNED* alleles as described^2^. The six offspring of the genome-edited polled bull (RC.calves1–6) were heterozygous for *POLLED* (P_c_p). The Horned Hereford control calves (HH.calves1–3) were homozygous horned (pp, Fig. 3 and Supplementary Fig. 1) as were the offspring of the Holstein sire (data not shown). The Horned Hereford cows had their horns removed physically, which is why no horns are visible in Fig. 2. Records for RC.dam1 indicate that she was disbudded along with the rest of her herdmates, but she is heterozygous P_C_p by PCR and therefore was naturally polled.

### Assessment of horned phenotype

By the 8-month exam, the purebred control Horned Hereford calves (HH.calves1–3) and the Holstein × Hereford calves (HO1.calves1–3) had developed horns, as expected. The calves sired by the genome-edited polled bull had not developed horns (Supplementary Fig. 2); however, the bull calves did develop small scurs (Supplementary Fig. 3). Scurs, corneous growths that can be of varying sizes and develop in the same area as horns but are not firmly attached to the skull, are a common occurrence in males heterozygous for *POLLED*^11^, so this result is not surprising or outside of normal parameters. The heifer calf did not develop scurs. Scurs map to a separate genetic locus from the *POLLED* locus, but the exact causal mutation remains unknown^12^. At the time of writing, the one remaining female calf is 23 months old and still has not developed horns.

### Assessment of fetal microchimerism

To evaluate whether fetal cells potentially crossed the placental barrier to the surrogate dams (fetal microchimerism), blood samples were taken from the dams 1 month before birth and at weeks 1, 2, 3, 4 and 5. DNA was extracted and assayed by quantitative PCR (qPCR) for *HORNED*, *POLLED*, a Y chromosome marker and a housekeeping gene (data not shown). All dams showed the presence of the *HORNED* allele, as expected. RC.dam1 showed the presence of the *HORNED* allele and the *POLLED* allele consistent with PCR results for this dam that indicate heterozygosity for the *POLLED* allele. None of the dams that carried male offspring showed the presence of the Y chromosome marker. The results did not show any transfer of the *POLLED* allele from the genome-edited polled sire offspring to the blood of the dams.

### Assessment of genomic variation

The genome-edited bull’s (RCI002) offspring were compared to matching controls with reference to the ARS-UCD1.2 bovine genome sequence (https://www.ncbi.nlm.nih.gov/assembly/GCF_002263795.1/), derived from a Hereford cow^13^, to determine whether the number of single nucleotide polymorphisms (SNPs), indels and Mendelian transmission rates were skewed in any of the study groups (GH.H versus H.H versus Ho.H).

### Variant calling and variant statistics

**Fig. 4:**
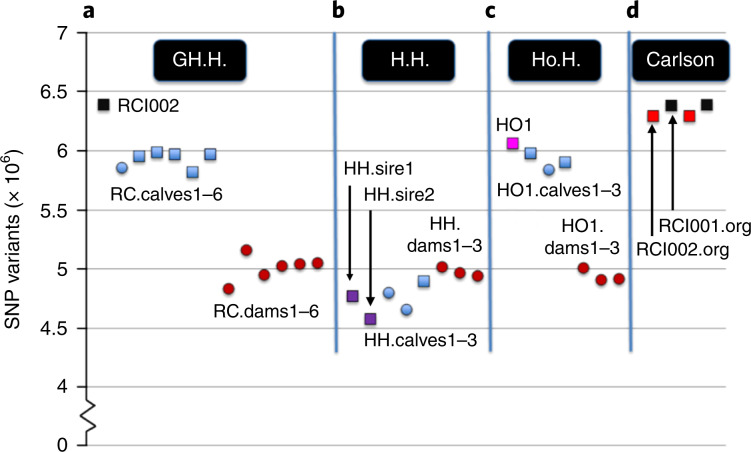
The number of SNP variants relative to the ARS-UCD1.2 bovine reference genome derived from a Hereford cow. The Hereford cow was L1 Dominette 01449 (ref. ^13^). Males (squares) and females (circles) are shown in four study groups. **a**, GH.H.: genome-edited polled bull (RCI002, black) was bred to Horned Hereford cows (RC.dams1–6, brown) and produced six polled offspring (RC.calves1–6, blue). **b**, H.H.: Horned Hereford bulls (HH.sire1 and HH sire23, purple) were bred to Horned Hereford cows (HH.dams1–3, brown) and produced three horned offspring (HH.calves1–3, blue). **c**, Ho.H.: Horned Holstein sire (HO1, pink) was bred to Horned Hereford cows (HO1.dams1–3, brown) by artificial insemination and produced three horned offspring (HO1.calves1–3, blue). **d**, Carlson: original sequences of cell lines (CL2122.org (black) and CL2120.org (red)) that were edited to produce bulls, RCI002.org (black) and RCI001.org (red), respectively, as reported by Carlson et al.^2^. RCI002 and RCI002.org are data from the same genome-edited bull sequenced by two different sequencing laboratories in different years.

GATK variant calling initially identified 17,758,947 variants. A subsequent quality filtration identified 14,155,980 variants as trusted. The numbers of variants (in the range of 4–7 million SNPs (Fig. 4) and 80,000–100,000 indels per individual) were comparable in all animals. There was an obvious result of fewer variants found when comparing the sequence of purebred Horned Herefords (H.H family) to the reference Hereford genome, as compared to sequences from purebred Holstein (HO1) or the Holstein cross (RCI002) bull, and offspring sired by these bulls (Fig. 4).

### Assessment of Mendelian errors

Biallelic variants (14,084,653) achieved a 99.8% genotyping rate and were included in further analyses. Another subset of variants was also selected by exclusion of 218,070 variants with genotype rate <95% and 2,537,388 variants with minor allele frequency <5%. The breakdown of heterozygous, compound heterozygous and homozygous mutants for each animal as compared to the reference genome is detailed in Supplementary Table 1. Four families with 12 meiotic divisions were tested for the number of errors according to the expected rate of Mendelian transmission (Table 1). With both datasets, the average rate of the errors in each meiotic division was 1.0% per variant (±0.2) with insignificant differences between the three studied groups (two one-way analysis of variance (ANOVA) d.f. = 2; *P* = 0.078, *F* = 3.43; *P* = 0.149, *F* = 2.369). Mendelian error rates in 10 kilobase regions accounting for a high proportion of inherited errors did not differ in range among the study families (Supplementary Fig. 4). ANOVA for the average error rates per study group (d.f. = 2, *F* = 61.101) showed no difference between GH.H. and Ho.H. groups (*P* = 0.897); however, both groups were significantly different from the H.H. group (*P* < 0.001; Supplementary Fig. 4). The 171 regions with consistently high error rates (>1 error per kb) in all three study groups were most prevalent on Chromosomes 12 and 23, and are listed in Supplementary Table 2.

**Table 1:** Mendelian error rates of *n* = 12 biologically independent sire/dam/offspring trios in four families

Study group	Family	Sire	Dam	Offspring	*N* errors	Percentage per variant	Percentage per individual	Sequencing coverage (×)		
								Sire	Dam	Offspring
GH.H.	1	RCI002	RC.dam1	RC.calf1	169,672	1.2	0.4	20.2	17.5	20.1
			RC.dam2	RC.calf2	158,736	1.1	0.4	20.2	18.8	19.5
			RC.dam3	RC.calf3	126,192	0.9	0.3	20.2	25.9	22.9
			RC.dam4	RC.calf4	143,283	1.0	0.3	20.2	19.8	21.3
			RC.dam5	RC.calf5	174,909	1.2	0.4	20.2	20.7	16.5
			RC.dam6	RC.calf6	137,177	1.0	0.3	20.2	18.7	26.7
H.H.	2	HH.sire1	HH.dam1	HH.calf1	125,899	0.9	0.3	17.4	20.6	21.4
	3	HH.sire23	HH.dam2	HH.calf2	120,690	0.9	0.3	22.2	17.5	19.4
			HH.dam3	HH.calf3	117,590	0.8	0.3	22.2	19.9	19.6
Ho.H.	4	HO1	Ho1.dam1	Ho1.calf1	141,404	1.0	0.3	19.6	20.0	21.7
			Ho1.dam2	Ho1.calf2	143,436	1.0	0.3	19.6	24.9	18.8
			Ho1.dam3	Ho1.calf3	184,300	1.3	0.4	19.6	15.9	17.2

*N* errors, number of Mendelian errors (offspring not concordant with parental genotypes) when comparing 14,084,653 variants, unfiltered for low genotyping rate or minor allele frequency; percentage per variant, percentage probability of a Mendelian error for each biallelic variant in a trio; percentage per individual, percentage probability of a Mendelian error for each biallelic variant in each individual. WGS coverage is shown for each individual.

### Assessment of insertion stability

**Fig. 5:**
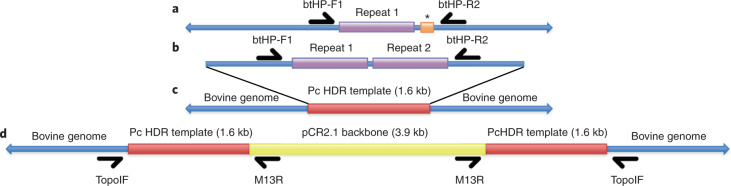
The alleles of the bovine *POLLED* locus. **a**,**b**, Difference between the wild type *HORNED* allele (**a**) and naturally occurring P_C_ *POLLED* allele (**b**) within the 1.6 kb HDR template sequence (Carlson et al.^2^) at the *POLLED* locus. The 212-bp repeat sequence (purple) is duplicated in the naturally occurring P_C_ *POLLED* allele and replaces the 10-bp (CTGGTATTCT) orange sequence (*) in the wild type *HORNED* allele. btHP-F1/btHP-R2 are PCR primers used by Carlson et al.^2^ and for our screening PCR in Fig. 3. **c**,**d**, The genome-edited bull RCI002 was a compound heterozygote carrying allele (**c**) the exact same sequence as the naturally occurring P_C_ *POLLED* allele and allele (**d**) that included both the pCR2.1 plasmid sequence (yellow) and a duplication of the Pc HDR template (red). topoIF/M13R and M13F/topoIR are PCR primer pairs.

A sequence baiting approach was used to investigate whether the 212 base pair repeat of the P_C_ *POLLED* allele was inserted anywhere in the genome other than the expected position. The sequence inserted in the correct location is expected to cause a duplication of an internal 5′ 212 bp in the cattle reference genome (Fig. 5a,b). If the sequence is appropriately inserted in, and only in, the expected position, all reads generated from the sequence of this insertion locus should be categorized into one of three classes when mapped back to the ARS-UCD1.2 bovine reference genome sequence: (1) reads mapping perfectly to the internal repeat or its 5′ junction with the reference genome, (2) reads mapping to the 3’ end of the internal repeat with a 16-bp deletion and (3) reads mapping with supplementary alignment to this locus but align perfectly over the junction between the two repeats in the reference genome sequence amended to have the insertion sequence (Fig. 5c). In this approach, we selected any sequence that shared at least 25 bp of the 212 bp of the P_C_ polled allele to find any possible degenerate or chimeric versions of the insertion sequence.

The sequence baiting approach found that all reads generated from the insertion sequence and the surrounding edges matched one of the three expected classes, with the exception of a single read. That read only mapped to the original and expected loci with supplementary alignments. Revised exact alignment of the read showed that it belonged to the third category above, but had many sequencing errors that prevented the direct alignment to the expected locus (Supplementary Fig. 5). Only those animals carrying one or two copies of the P_C_ *POLLED* allele had reads that aligned perfectly to class c, meaning they aligned around the insertion position in the ARS-UCD1.2 bovine reference genome sequence at the predicted insertion sequence. The P_C_ *POLLED* allele did not insert anywhere in the genome other than the expected position.

### Assessment for the presence of plasmid sequence

Alignment of the short read genomic sequences to the donor plasmid pCR 2.1 (Life Technologies) revealed that in addition to the intended P_C_ *POLLED* allele, the entire 3.9 kb pCR 2.1 plasmid sequence and an additional copy of the P_C_ HDR template introgressed into one of the alleles of the polled bull (RCI002, Fig. 5d). This was stably transmitted to four of the six offspring (RC.calf1,4,5,6, Supplementary Fig. 6). Further PCR-based analysis and Sanger sequencing confirmed the presence of this plasmid insert in these, and only these, five animals. Long read Nanopore WGS generated ~4.3 million reads containing ~37 million nucleotides to achieve 13.7× coverage of the RCI002 genome. The *k*-mer baiting approach was used to select any reads with similarity to the pCR2.1 plasmid sequence or the P_C_ HDR template sequence. The reads were aligned to two predicted alleles of the edited ARS-UCD reference assembly (Fig. 5c,d). The mapping results confirmed the presence of the two alleles with eight reads supporting the allele carrying the pCR2.1 plasmid sequence and a duplication of the P_C_ HDR template and 12 reads supporting the allele having the exact sequence of the naturally occurring P_C_ *POLLED* allele.

## Discussion

Our report presents a detailed analysis of the offspring of a bull that was genome edited to be homozygous for the P_C_ *POLLED* allele. This intentional alteration involved the use of a P_C_ HDR template DNA sequence in a plasmid to guide HDR of a TALEN-mediated double-stranded break at the *POLLED* locus. The six F1 offspring all inherited this dominant allele from their sire and were phenotypically polled, as predicted. Furthermore, we found that the bull was a compound heterozygote with one naturally occurring P_C_ allele, and one allele including donor plasmid sequence and a duplication of the P_C_ HDR template. Using a single-stranded oligodeoxynucleotide (ssODN) or DNA (ssDNA) repair template, rather than a donor plasmid, would eliminate the possibility of such a plasmid backbone integration. Other than this finding, there were no remarkable or unexpected findings in the phenotypes or genomes of the offspring with the exception of a single bull calf with one undescended testicle. The genome-edited bull RCI002 also had an undescended testicle. This trait, known as cryptorchidism, has moderate heritability. The polled phenotype is not known to be associated with cryptorchidism, although some breeds (Polled Hereford and Shorthorn) are at a greater risk for cryptorchidism^14^.

The bioinformatics analyses revealed that the P_C_ allele was stably inherited, was at the expected location in the genome, and that the Mendelian error rate did not differ between the genome-edited offspring and contemporary controls. It also underscored the important impact that breed has on genome variation. The Horned Hereford cattle in this study had 1–1.5 million fewer SNP variants relative to the Hereford ARS-UCD1.2 bovine reference genome sequence than the dairy breeds (Fig. 4).

A recent study of WGS data from 2,703 individual cattle in the 1,000 Bull Genomes Project revealed more than 86.5 million differences (variants) between different breeds of cattle^15^. These variants included 2.5 million insertions and deletions of one or more bp, and 84 million single nucleotide variants. Another source of genomic variation is the 30–40 spontaneous de novo mutations (insertions, substitutions or deletions) that occur naturally every generation. For example, the single nucleotide variant de novo mutation rate (base pair per generation) is estimated to be 1.15 × 10^−8^ in goats^6^, 1.36 × 10^−8^ in sheep^5^ and 1.25 × 10^−8^ in cattle^16^, which are similar rates to estimates in humans^17^. In fact, these mutations are the fuel that drives both natural selection and the artificial selection programs practiced by animal breeders. This variation needs to be accounted for when considering genomic analysis to detect unintended alterations (for example, off-target alterations, unanticipated insertions, substitutions or deletions) as suggested by the FDA draft guidance no. 187, ‘Regulation of Intentionally Altered Genomic DNA in Animals’^8^. What remains uncertain is what level of off-target alterations is acceptable, or unacceptable, and the fact that there is no obvious way to differentiate between unintended alterations and spontaneously occurring insertions, substitutions, deletions and other unpredictable naturally occurring alterations. Additionally, it is unclear what unique risks are posed by editing-associated, unintentional, off-target DNA alterations in food animals that are not also equally posed by the even higher rate of naturally occurring background spontaneous de novo mutations.

A donor template plasmid sequence insertion was detected when the genomic sequences were aligned to the donor template pCR2.1 plasmid sequence. The plasmid and an additional copy of the P_C_ HDR template sequence had inserted adjacent to the intended alteration at the polled locus in one of the alleles carried by the genome-edited bull (Fig. 5d). This insertion was not identified when aligning the genomic sequences to the reference bovine genome^2^, nor was it detected when using the common M13F/R PCR primers, due to its integration orientation. The other allele carried by the bull was the intended naturally occurring P_C_ *POLLED* allele. These alleles segregated in the offspring, with four inheriting the allele with the plasmid sequence. Both alleles resulted in the hornless phenotype, and no other phenotypic effects were evident in either the bull or the four offspring that inherited the allele with plasmid sequence. This finding reinforces the need to screen for plasmid sequence when genome editing involves a plasmid containing the HDR repair template, as has been done in other studies^7^. Such screening is routinely done in plant breeding, where conventional genome editing typically involves the delivery and integration into the host genome of DNA cassettes encoding editing components. Final edited-plant products are typically null-segregants containing the intended genomic alteration but none of the plasmid DNA from the editing cassettes^18^. Ideally, screening for plasmid sequences would be undertaken before an animal is produced; however, this is challenging when gene editing components are being delivered via CPI into one-cell zygotes, as biopsying embryos before embryo transfer decreases their viability and results from trophectoderm biopsies may not reflect all cells of the animal due to mosaicism^7^.

Our results largely agree with the two other studies in food animals that looked at trio-based WGS of genome-edited (CRISPR/Cas9) sheep and goats^5,6^. Both of these papers examined targeted gene knockouts where the nuclease introduces a site-directed double-strand break, which is repaired by the cell’s inherently error-prone DNA repair mechanisms, and hence no HDR plasmid was involved. These analyses, which involved sequencing father/mother/offspring trios, found that rates of de novo variants were negligible compared to the average spontaneous germline de novo mutation rate. The sheep study did reveal a single 2.4 kb inversion in one of 54 founder animals, which the authors postulated was due to a double-stranded cleavage at two single gRNA target sites. These findings are consistent with previous CRISPR/Cas9 off-target studies in humans^19,20^, monkeys^21^ and rodents^22–24^, which suggest the rate of Cas9-mediated mutagenesis is not distinguishable from the background de novo mutation rate.

In addition to questions about genomic variation, concerns have historically been voiced that genetically engineered offspring could pass exogenous genetic information to their dams during gestation and birth. Surrogate dams that have given birth to genetically engineered offspring are therefore treated as if they themselves are genetically engineered, due to a concern that fetal cells can cross the placental barrier and reside in the mother (fetal-maternal microchimerism). This precludes their entry into the food supply, and requires that these animals and their biological products (including milk) be disposed of by incineration, burial or composting^25^. This further increases the cost and decreases the economic feasibility of performing experimental work with recombinant DNA technologies, including genome editing. We did not find any evidence of fetal microchimerism for any of the loci tested by qPCR in any of the dams. The hazard associated with fetal microchimerism when considering a genomic alteration that could have been achieved with conventional breeding is difficult to define. No notable differences were detected between the dams of the offspring from the genome-edited polled sire as compared to the dams bred to the control sires, and there was no indication that any potentially hazardous changes had occurred to the dams as a result of gestating offspring from a genome-edited polled bull.

Plants and animals produced using conventional breeding methods are not routinely evaluated for unintended effects at the molecular level^26^. According to the White House Office of Science and Technology Policy, federal oversight of the products of biotechnology “will be exercised only where the risk posed by the introduction is unreasonable, that is, when the value of the reduction in risk obtained by additional oversight is greater than the cost thereby imposed. The extent and type of oversight measure(s) will thus be commensurate with the gravity and type of risk being addressed, the costs of alternative oversight options, and the effect of additional oversight on existing safety incentives”^27^.

The advent of genome editing offers an opportunity to rethink the regulatory approach to the products of biotechnology, and a number of authors have proposed that the trigger for additional regulatory review should be any novel product hazards/risks, weighed against the resulting benefits^28–36^. The FDA has regulated genetically engineered animals carrying rDNA constructs as new animal drugs since 2009 (ref. ^25^). The FDA’s regulatory authority over new animal drugs comes from the Federal Food, Drug and Cosmetic Act (FD&C Act). The definition of a drug, in section 201(g) of the FD&C Act, includes “articles intended for use in the diagnosis, cure, mitigation, treatment, or prevention of disease in man or other animals”; and “articles (other than food) intended to affect the structure or any function of the body of man or other animals”^8^. Until now, only one engineered food animal, the AquAdvantage salmon, has managed to successfully navigate this multigenerational premarket regulatory approval process; a process that took more than a decade and cost millions of dollars^37^.

According to the FDA’s 2017 draft guidance^8^, developers of genome-edited animals should fully characterize the site of the intentional alteration and any unintended alterations (for example, off-target alterations, unanticipated insertions, substitutions or deletions), particularly for coding or regulatory regions. Moreover, the types of analyses outlined in this paper are required for each specific genomic alteration, as “each specific genomic alteration is considered to be a separate new animal drug subject to new animal drug approval”^8^. Additionally, the guidance suggests developers should perform studies showing that genotypic alterations are durable, meaning that the altered genomic DNA is stably inherited. For phenotypic durability, data showing consistency of the expressed trait over multiple generations is recommended. It is also recommended that data on inheritance be collected from at least two generations, preferably more, and at least two of the sampling points should be from noncontiguous generations (for example, F1 and F3).

We present data on one generation, the F1, in this study. Realistically, multigenerational studies in large livestock species with long generation intervals such as cattle make such studies exceptionally expensive in terms of both time investment and cost, especially when offspring are not allowed to enter the food supply. In our experiment, the genome-edited bulls were born in April 2015, and four years later we have F1 data. The female progeny is now pregnant, and we expect to be able to collect milk from her sometime in 2020. The high costs associated with mandatory multigenerational phenotypic and genomic studies for intentional genomic alterations in livestock will likely preclude many public sector researchers, and dissuade small companies, from pursuing food animal genome editing research and applications.

The FDA’s proposed new animal drug approach to the regulation of intentional genomic alterations introduced into food animals by editing would appear to be disproportionate to the gravity and type of risk being addressed, especially for alterations that could have been achieved using conventional breeding. The results from our study will inform the discussion regarding the need for such detailed and costly analyses. It is unlikely that animal genetic providers are in a position to sustain the high costs associated with new animal drug approvals for each specific genomic alteration. This may forestall the use of genome editing technology in food animal breeding programs, despite the valuable contribution this technology could make to animal welfare and health.

## Methods

### Breeding, animal management and veterinary exams

All animals were maintained at the UC Davis Animal Science Beef Barn and managed by facility staff according to approved protocols. The UC Davis veterinary hospital large animal clinic provided veterinary care.

Horned Hereford cows that are part of the UC Davis Animal Science teaching herd were estrus-synchronized according to standard protocols. Semen collection from RCI002 and subsequent artificial insemination were performed by veterinarians from the UC Davis veterinary hospital large animal clinic under standard procedures. Semen straws were purchased from commercial sources for the Horned Hereford and Holstein bulls. Pregnancies were monitored by UC Davis veterinarians by ultrasound. Experimental procedures were reviewed and approved by the UC Davis Institutional Animal Care and Use Committee (protocol no. 18855). All calves were monitored and handled by university staff according to standard facility operating procedures.

Blood samples were collected by venipuncture from coccygeal veins for adult animals and from jugular veins for initial blood sample collection from calves. Whole blood (5–10 ml) was collected in EDTA vacutainers (Becton Dickinson) by a veterinarian from the UC Davis veterinary hospital large animal clinic. Complete blood counts and chemistry panels were conducted and analyzed at the UC Davis veterinary hospital using determined reference intervals for cattle. DNA samples were extracted as described below and submitted to the UC Davis Veterinary Genetics Laboratory for parentage verification testing. Additional parentage verification for one animal was performed using the SeekSire test available through GeneSeek.

### DNA extraction, library preparation and WGS

Whole blood samples were collected as described above from the 28 individuals that were sequenced (Fig. 1) and centrifuged at 2,000 r.p.m. in a Sorvall tabletop centrifuge for 10 min to isolate white blood cells. DNA was extracted from the buffy coat using the DNeasy Blood and Tissue Kit (Qiagen) according to the manufacturer’s instructions, with the modification of double the amount of proteinase K and buffer AL (as suggested by Qiagen technical support). DNA was extracted from 50 μl of isolated white blood cells and eluted into 50 μl of buffer AE. Samples used for WGS were eluted in 50 μl of buffer EB. DNA concentrations were determined using a NanoDrop 2000 spectrophotometer (Thermo Fisher Scientific).

DNA samples were submitted to the QB3 Vincent J. Coates Genomics Sequencing Laboratory at UC Berkeley for next generation library construction and WGS. Samples were sequenced on an Illumina HiSeq4000 with paired end, 150 base pair reads. The sequencing covered the whole genomes of 28 cattle with ~5.7 billion paired-end fragments with an average ~200 million per animal to achieve ~20× coverage (±2.6). On average, 99% of input paired reads survived the quality-trimming step. The mapping rate to the reference genome was ~99% per animal with ~93% of the read pairs mapping appropriately as expected for their fragment sizes.

### DNA extraction, library preparation and nanopore resequencing

Liver (94 mg) from the genome-edited bull, RCI002, was incubated overnight in lysis buffer (0.2 M NaCl, 0.1 M Tris pH 8.5, 5 mM EDTA, 0.2% SDS) with 40 U Proteinase K (New England Biolabs) at 55 °C. Two extractions were performed using phenol:chloroform:isoamyl alcohol (25:24:1), and one extraction using chloroform. The DNA was precipitated using 2.5 volumes 100% ethanol and 0.1 volume of 5 M NH_4_OAc. The DNA was spooled and placed into 70% ethanol, spun at 7,600*g*, 5 min at 4 °C, dried and resuspended in EB buffer (Qiagen), briefly heated at 65 °C for 5 min then incubated overnight at room temperature with gentle agitation. Quantification of DNA was performed using a Qubit Fluorometer (Thermo Fisher Scientific).

The integrity of the high-molecular-weight DNA samples was verified on a Pippin Pulse gel electrophoresis system (Sage Sciences). The DNA was then sheared to an average size of 50 kb using a Megaruptor instrument (Diagenode) and verified on a Pippin Pulse gel. A sequencing library was prepared starting with 2 µg of sheared DNA using the ligation sequencing kit SQK-LSK109 (Oxford Nanopore Technologies) following instructions of the manufacturer with the exception of extended incubation times for DNA damage repair, end repair, ligation and bead elution. Then, 30 fmol of the final library was loaded on the PromethION flowcell R9.4.1 (Oxford Nanopore Technologies) and the data was collected for 64 h. Basecalling was performed live on the compute module using MinKNOW v.19.01.6 (Oxford Nanopore Technologies). A *k*-mer baiting approach with the pCR2.1 plasmid sequence and the Pc HDR repair sequence was used to select any reads with similarity to these sequences.

### Assessment of phylogenetic distances

The dendrogram (Fig. 1) was constructed to represent the IBS distance between the sequenced animals. To perform this analysis, all detected variants were filtered to remove those failing to genotype in more than 5% of all sequenced subjects as well as those with minor allele frequency less than 5%. Remaining variants were subjected to linkage disequilibrium-based pruning on a threshold of variance inflation factor equals two. Pruning recursively removed SNPs within a sliding window of 50 SNPs, with a window step size of five SNPs. The distance matrix was constructed using the ‘--distance 1-ibs’ function of PLINK 1.9 (www.cog-genomics.org/plink/1.9/)^38^ and plotted as a dendrogram using the ‘ape’ package in R^39^.

### Evaluation of insertion stability

To find any degenerate or chimeric version of the insertion sequence, we selected any sequence read that shared any stretch of 25 nucleotides with the 212 bp of the P_C_ polled allele. The reads were aligned against the ARS-UCD1.2 reference assembly (https://www.ncbi.nlm.nih.gov/assembly/GCF_002263795.1/) and the expected edited version based on the Pc HDR template sequence. Both alignment steps were done using BWA v.0.7.17 (ref. ^40^).

### Assessment of genomic variation

Quality assessment of the sequencing reads used FastQC v.0.11.7 (https://www.bioinformatics.babraham.ac.uk/projects/fastqc/) and multiqc v.1.0 (ref. ^41^). Trimmomatic software (v.0.36)^42^ removed the adapters and low quality sequences. High quality reads were aligned to the bovine reference genome ARS-UCD1.2 (https://www.ncbi.nlm.nih.gov/assembly/GCF_002263795.1/) using the BWA-MEM algorithm of the BWA software package (v.0.7.7)^40^. Replicate samples were merged using the MergeSamFiles tool and duplicate reads were excluded using the MarkDuplicates tool from the Picard software package v.2.18.1 (http://broadinstitute.github.io/picard). Variant calling was performed with the GATK v.4.0.9.0 (ref. ^43^) using the best practice for germline short variant discovery (https://software.broadinstitute.org/gatk/best-practices/workflow?id = 11146). The BaseRecalib and ApplyBQSR tools of GATK were used to recalibrate the quality scores of sequencing reads using the known variants from the Ensembl variation database (release 94)^44^. The HaplotypeCaller tool of GATK was used for joint genotyping across all sequenced samples. Candidate variants were filtered using the following thresholds: QualByDepth (QD) <2.0, FisherStrand (FS) >60.0, StrandOddsRatio (SOR) >4.0, ReadPosRankSum <−8.0 and depth of coverage (DP) >3,105 for both SNPs and indels, RMSMappingQuality (MQ) <40.0, MQRankSum <−12.5 for SNPs and InbreedingCoeff <−0.8 for indels. All samples were compared regarding the sequence quality and coverage, mapping rates and variant filtration statistics. To identify likely misassembled regions that can account for a higher than expected proportion of inherited errors, intervals of 10 kb along the whole genome were examined, and intervals with a high rate of Mendelian errors were identified. Those with fewer than or equal to ten errors among all animals were excluded (217,619), leaving 4,438 error-prone intervals. The number of errors in these intervals was then compared among the 12 offspring.

### Assessment of plasmid sequence

Short read genomic sequences for each sample were aligned to the donor plasmid pCR 2.1. PCR was used to analyze the orientation of the pCR 2.1 plasmid and confirm the duplication of the HDR template. Primers were developed using Primer3 (ref. ^45^) (Supplementary Table 4) to amplify the region flanking the polled locus. The topoIF primer was designed targeting the region upstream of the 5′ end of the polled locus and was paired with the M13R primer for PCR. The topoIR primer was designed targeting the region downstream of the 3′ end of the polled locus and was paired with the M13F primer for PCR (Fig. 5). PCR was performed on a SimpliAmp Thermal Cycler (Applied Biosystems) with 12.5 μl GoTaq Green Master Mix (Promega Biosciences LLC), 9.5 μl of water, 1 μl of each primer at 10 mM and 1 μl of DNA for 5 min at 95 °C, 35 cycles of 30 s at 95 °C, 30 s at 54 °C for topoIF/M13R or 57 °C for M13F/topoIR (Supplementary Table 3) and 2.5 min at 72 °C, followed by 10 min at 72 °C. Products were visualized on a 1% agarose gel using a ChemiDoc-ItTS2 Imager (UVP, LLC), purified using the QIAquick PCR Purification Kit (Qiagen, Inc.) and Sanger sequenced (GeneWiz).

### Assessment of fetal microchimerism

DNA samples extracted as described above were submitted to the UC Davis School of Veterinary Medicine Real-time PCR Research and Diagnostics Core Facility for qPCR and subsequent analysis. For each target gene, two primers and an internal, fluorescent labeled TaqMan probe (5′ end, reporter dye FAM (6-carboxyflourescein), 3′ end, nonfluorescent quencher dye) were designed using Primer Express software (Applied Biosystems) (Supplementary Table 5). TaqMan PCR systems were validated using defined protocols^46^.

TaqMan PCR systems were validated using ten-fold dilutions of DNA testing positive for the target genes. The dilutions were analyzed in triplicate and a standard curve plotted against the dilutions. The slope (*s*) of the standard curve was used to calculate amplification efficiencies using the formula *E* = 10^(−1/*s*)^ − 1. To pass validation, all efficiencies had to be greater than 90%.

Each qPCR reaction contained 400 nM primers and 80 nM probe, commercially available PCR master mix (cat. no. 431815, TaqMan Universal PCR Master Mix, Thermo Fisher Scientific) containing 10 mM Tris-HCl (pH 8.3), 50 mM KCl, 5 mM MgCl2, 2.5 mM deoxynucleotide triphosphates, 0.625 U AmpliTaq Gold DNA polymerase per reaction, 0.25 U AmpErase UNG per reaction and 5 μl of DNA at a 1:5 dilution. qPCR was performed using an automated fluorometer (ABI PRISM 7900 HTA FAST, Thermo Fisher Scientific). The following amplification conditions were used: 2 min at 50 °C, 10 min at 95 °C, 40 cycles of 15 s at 95 °C and 60 s at 60 °C. Fluorescent signals were collected during the annealing phase and Cq values extracted with a threshold of 0.1 and baseline values of 3–15.

### Statistics

One-way ANOVA tests were done using the ANOVA function of the Stats Package in R v.3.5.1.

### Reporting summary

Further information on research design is available in the Nature Research Reporting Summary linked to this article.

## Online content

Any methods, additional references, Nature Research reporting summaries, source data, statements of code and data availability and associated accession codes are available at 10.1038/s41587-019-0266-0.

## Supplementary Information

### Integrated supplementary information

Supplementary Figure 1 Original scan of the PCR results depicted in Figure 3. PCR products are shown for the genotyping of the polled locus for offspring of the genome-edited bull (first 6 lanes) and the Horned Hereford control offspring (next three lanes), with homozygous polled (P_C_P_C_; 591 bp), homozygous horned (pp; 389 bp), and negative PCR controls in the last 3 lanes. The molecular weight marker is in the far left lane.

Supplementary Figure 2 Calves in this study at 8 months of age. RCI.calf1-6 (top row) did not develop horns, whereas HH.calves1–3 and HO1.calves1–3 (bottom row) did develop horns.

Supplementary Figure 3 Pictures of scurs on two of the five male offspring of the genome-edited bull. All five heterozygous males showed scurs of varying sizes on one or both sides. Shown are bull calves RCI.calf5 (L) and RCI.calf6 (R).

Supplementary Figure 4 Box plot of the number of Mendelian errors based the analysis of *n* = 12 biologically independent sire/dam/offspring trios at 4,438 10 kb regions of the genome with a high proportion of inherited errors. Zero errors was set to 0.5 to allow for the log2 conversion. RC.calves1–6 are the offspring of the genome edited, polled bull. HH.calves1–3 are the Horned Hereford control offspring. HO1.calves1–3 are the control offspring from the Holstein bull. The figure was generated using the default parameters of the boxplot function in the package graphics version 3.5.1. Box-and-whisker plot: center line, median; bottom of box, 25% quartile (Q1); top of box, 75% quartile (Q3); whiskers, Q1 - 1.5 IQR and Q3 + 1.5 IQR where IQR is the interquartile range = Q3 - Q1.

Supplementary Figure 5 Reads from heterozygous and homozygous P_C_ cattle shown mapping over the junction between the two repeats in the edited ARS-UCD1.2 bovine reference genome sequence that has the insertion sequence. Note that although there are sequence variations indicated by colored dots, there is no consistent pattern suggesting sequencing errors rather than induced mutations. One read (colored in purple) mapped unexpectedly with supplementary alignment. Revised exact alignment of the read showed it should have mapped to the insertion position but had many sequencing errors that prevented the direct alignment to the expected locus.

Supplementary Figure 6 Genomic sequence alignment to donor plasmid and P_C_ homology-directed repair (HDR) template. Alignment of the short-read genomic sequences to the pCR2.1 backbone (yellow) showed A) no read coverage across the backbone (n=23), or B) the presence of the plasmid in 5 of the animals (RCI002, RC.calf1, RC.calf4, RC.calf5, RC.calf6).

### Supplementary information

Supplementary Materials Supplementary Figs. 1–6 and Tables 1–5.

Reporting Summary

## Data Availability

WGS have been deposited in the NBCI Sequence Read Archive under BioProject PRJNA494431. Sequences from Carlson et al. are under BioProject PRJNA316122 (ref. ^2^). See Supplementary Table 3 for a full list of accession codes. Figures 1 and 4 and Table 1 are based on the raw data contained in the sequence data. There are no restrictions on data availability.

## Code availability

The code used in this study is available on GitHub at https://github.com/dib-lab/Bovine_seq.
