# Author Correction: Genomic and phenotypic analyses of six offspring of a genome-edited hornless bull

**DOI:** 10.1038/s41587-020-0423-5

**Published:** 2020-01-28

**Authors:** Amy E. Young, Tamer A. Mansour, Bret R. McNabb, Joseph R. Owen, Josephine F. Trott, C. Titus Brown, Alison L. Van Eenennaam

**Affiliations:** 1 Department of Animal Science, University of California, Davis, CA USA; 2 Department of Clinical Pathology, School of Medicine, University of Mansoura, Mansoura, Egypt; 3 Department of Population Health and Reproduction, School of Veterinary Medicine, University of California, Davis, CA USA

**Keywords:** Animal biotechnology, Agricultural genetics, Animal breeding

Correction to: *Nature Biotechnology* 10.1038/s41587-019-0266-0, published online 7 October 2019.

In the version of this article initially published online, the color of the red and black squares was reversed in Fig. 4d and the labels RCI001 and RCI002 were reversed in Supplementary Tables 1 and 3. The errors have been corrected in the print, PDF and HTML versions of the article.

