# Supplementary figures and images for: Genomic and phenotypic analyses of six offspring of a genome-edited hornless bull
Source: Nat Biotechnol. 2019 Oct 7;38(2):225–32. doi: 10.1038/s41587-019-0266-0 (PMC7007412; doi:10.1038/s41587-019-0266-0)

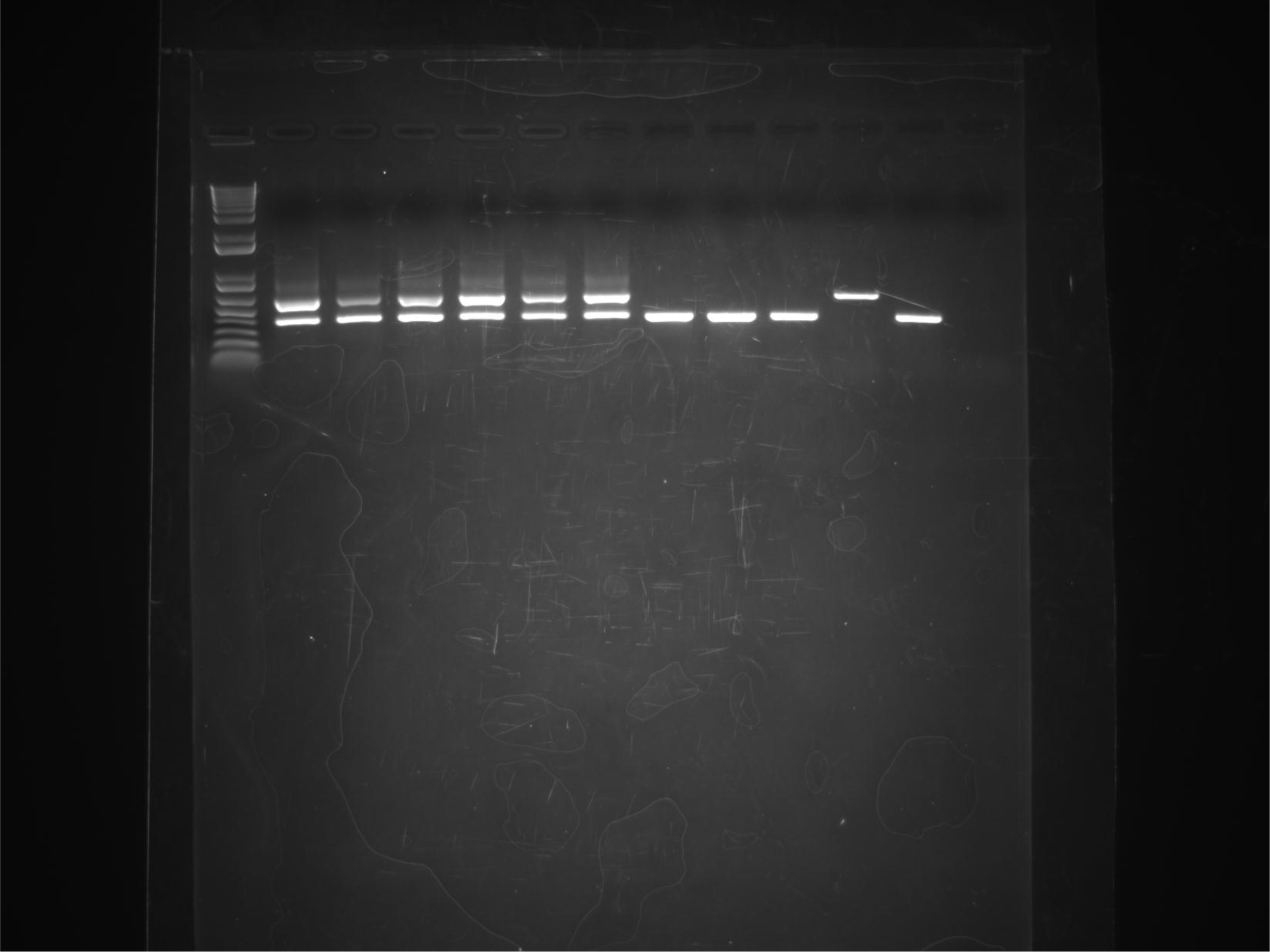

Supplement: Original scan of the PCR results depicted in Figure 3. — PCR products are shown for the genotyping of the polled locus for offspring of the genome-edited bull (first 6 lanes) and the Horned Hereford control offspring (next three lanes), with homozygous polled (PCPC; 591 bp), homozygous horned (pp; 389 bp), and negative PCR controls in the last 3 lanes. The molecular weight marker is in the far left lane. [file 41587_2019_266_Fig6_ESM.jpg]

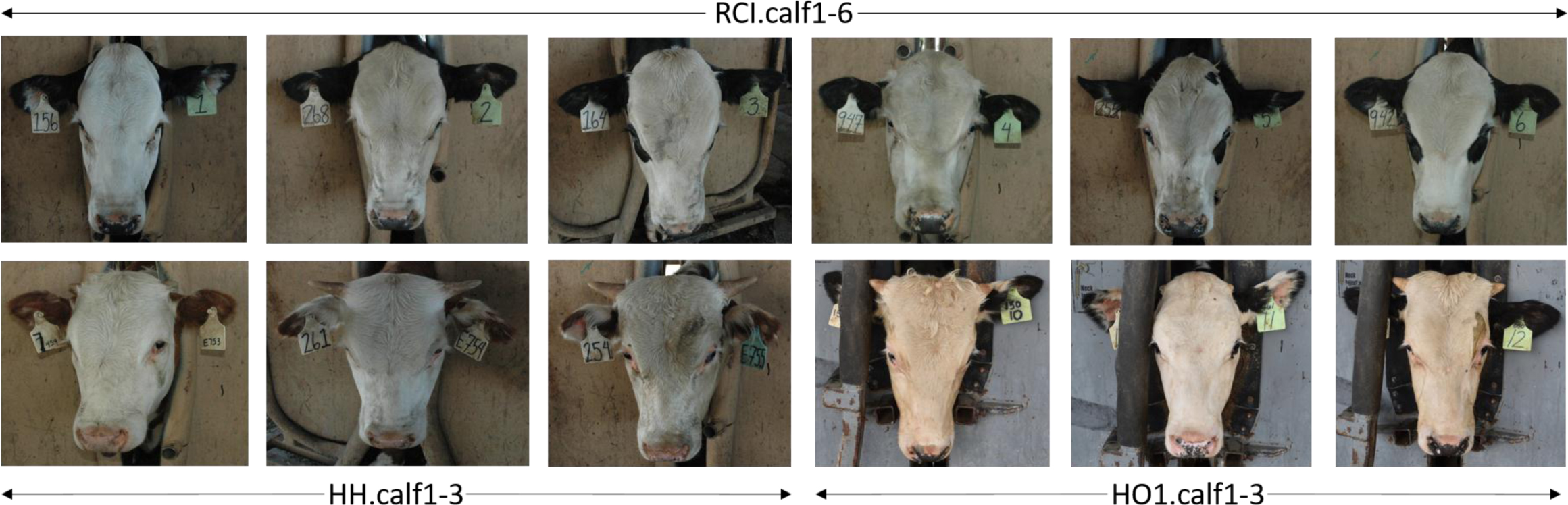

Supplement: Calves in this study at 8 months of age. — RCI.calf1-6 (top row) did not develop horns, whereas HH.calves1–3 and HO1.calves1–3 (bottom row) did develop horns. [file 41587_2019_266_Fig7_ESM.jpg]

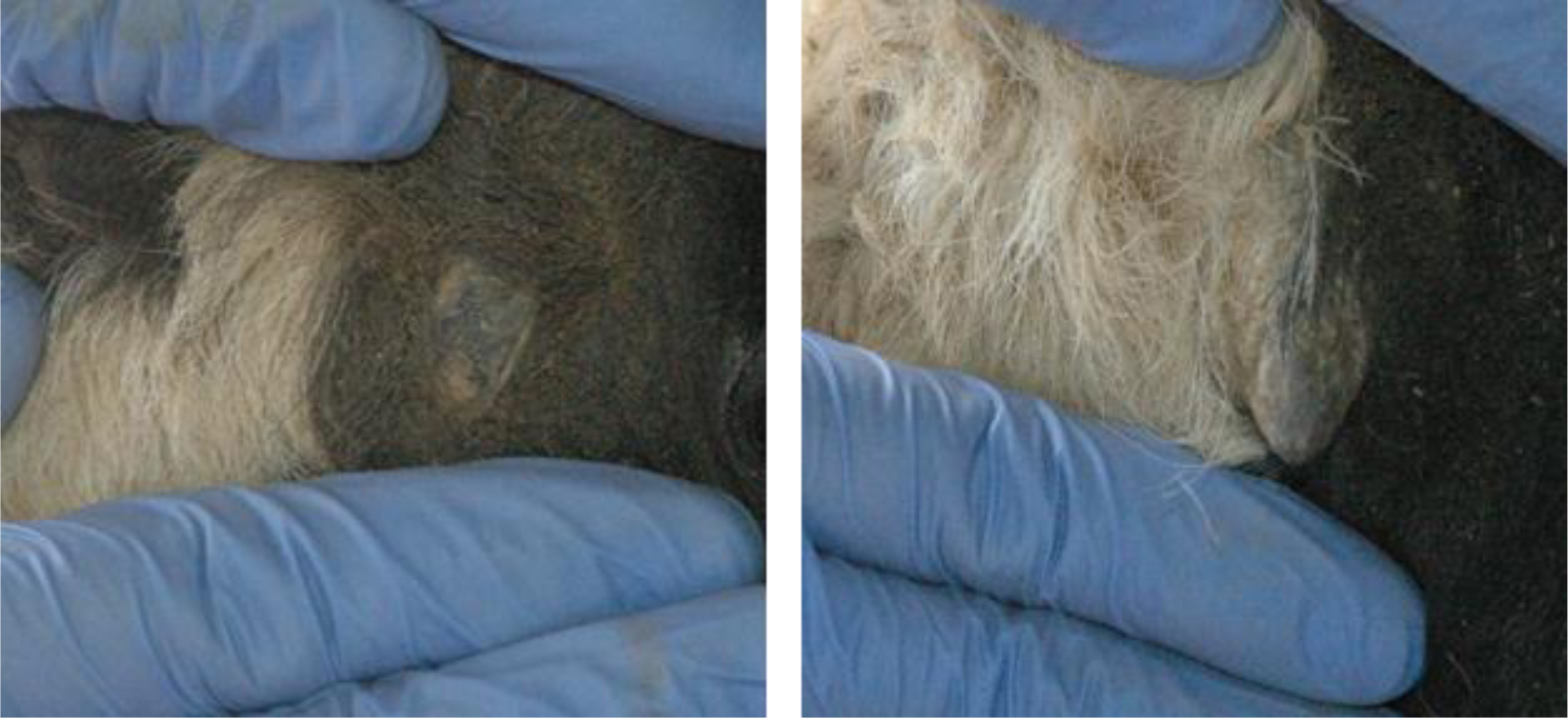

Supplement: Pictures of scurs on two of the five male offspring of the genome-edited bull. — All five heterozygous males showed scurs of varying sizes on one or both sides. Shown are bull calves RCI.calf5 (L) and RCI.calf6 (R). [file 41587_2019_266_Fig8_ESM.jpg]

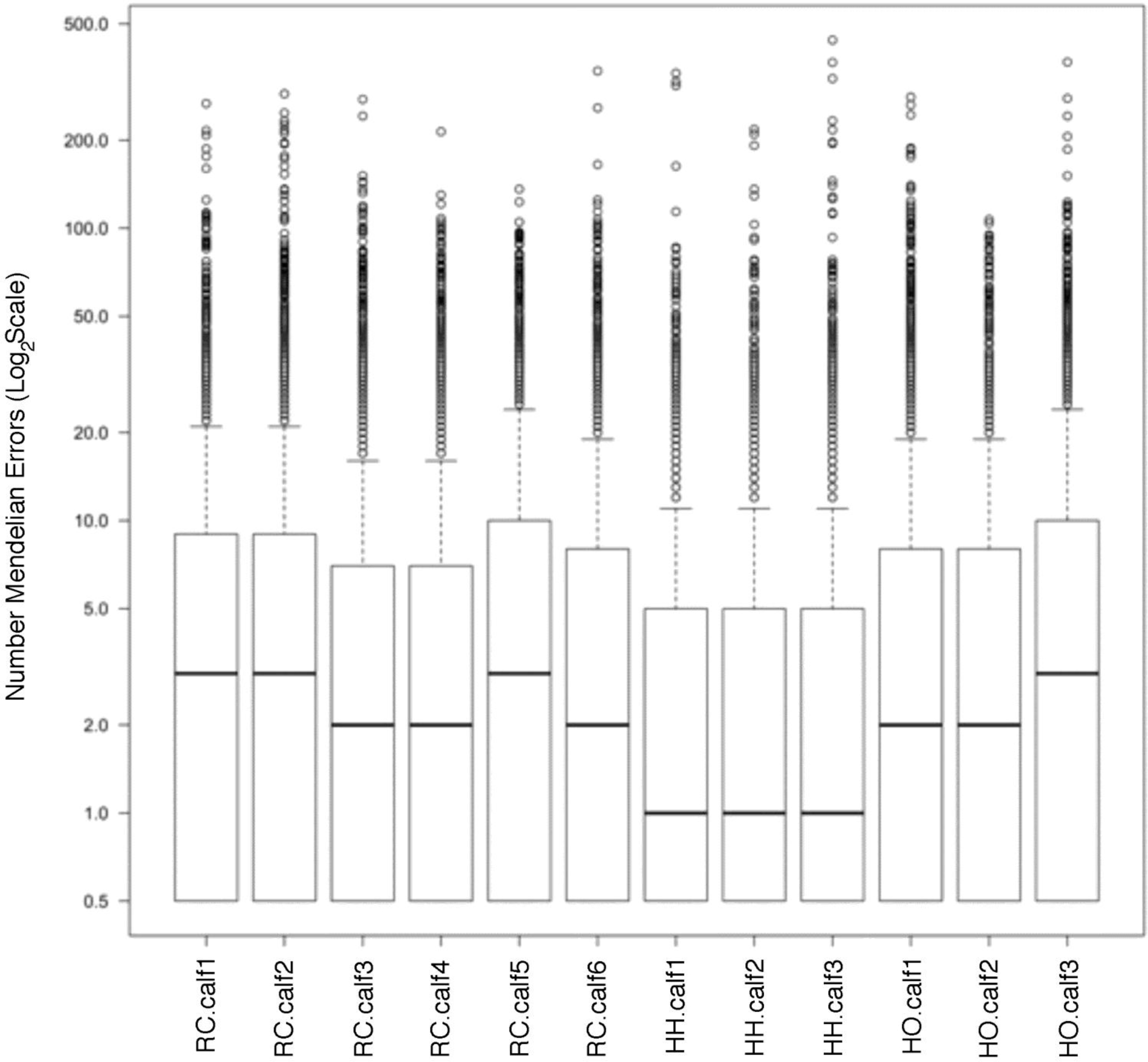

Supplement: Box plot of the number of Mendelian errors based the analysis of n = 12 biologically independent sire/dam/offspring trios at 4,438 10 kb regions of the genome with a high proportion of inherited errors. — Zero errors was set to 0.5 to allow for the log2 conversion. RC.calves1–6 are the offspring of the genome edited, polled bull. HH.calves1–3 are the Horned Hereford control offspring. HO1.calves1–3 are the control offspring from the Holstein bull. The figure was generated using the default parameters of the boxplot function in the package graphics version 3.5.1. Box-and-whisker plot: center line, median; bottom of box, 25% quartile (Q1); top of box, 75% quartile (Q3); whiskers, Q1 - 1.5 IQR and Q3 + 1.5 IQR where IQR is the interquartile range = Q3 - Q1. [file 41587_2019_266_Fig9_ESM.jpg]

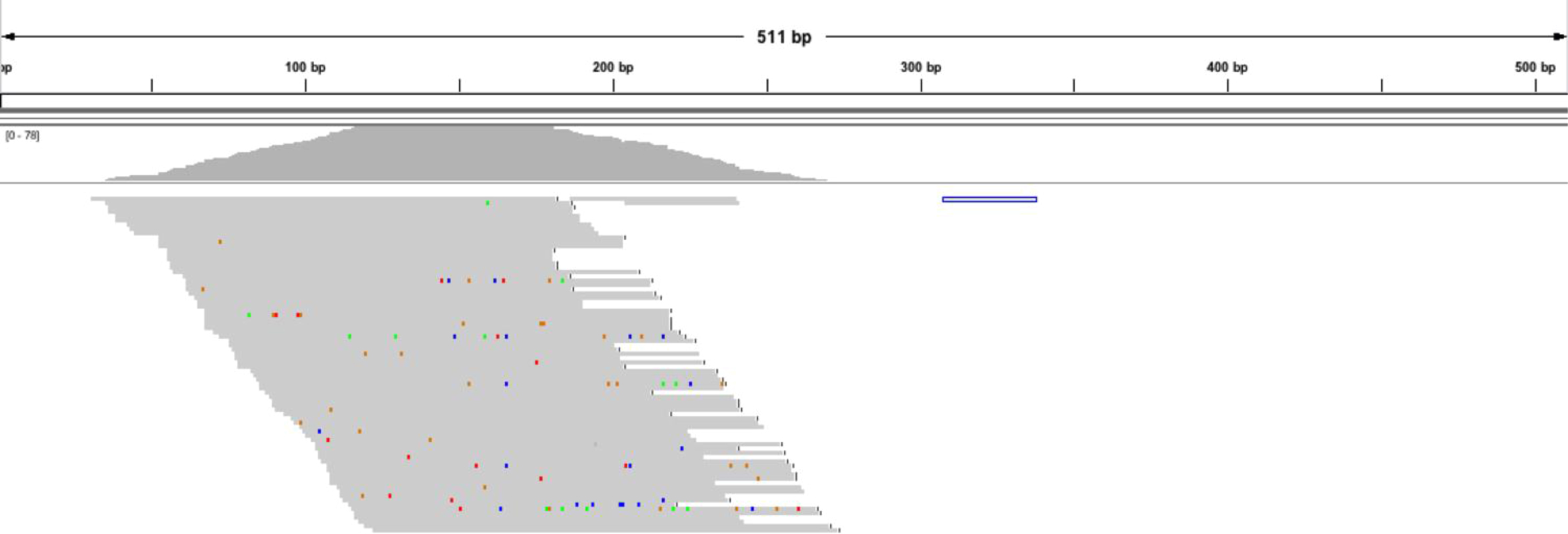

Supplement: Reads from heterozygous and homozygous PC cattle shown mapping over the junction between the two repeats in the edited ARS-UCD1.2 bovine reference genome sequence that has the insertion sequence. — Note that although there are sequence variations indicated by colored dots, there is no consistent pattern suggesting sequencing errors rather than induced mutations. One read (colored in purple) mapped unexpectedly with supplementary alignment. Revised exact alignment of the read showed it should have mapped to the insertion position but had many sequencing errors that prevented the direct alignment to the expected locus. [file 41587_2019_266_Fig10_ESM.jpg]

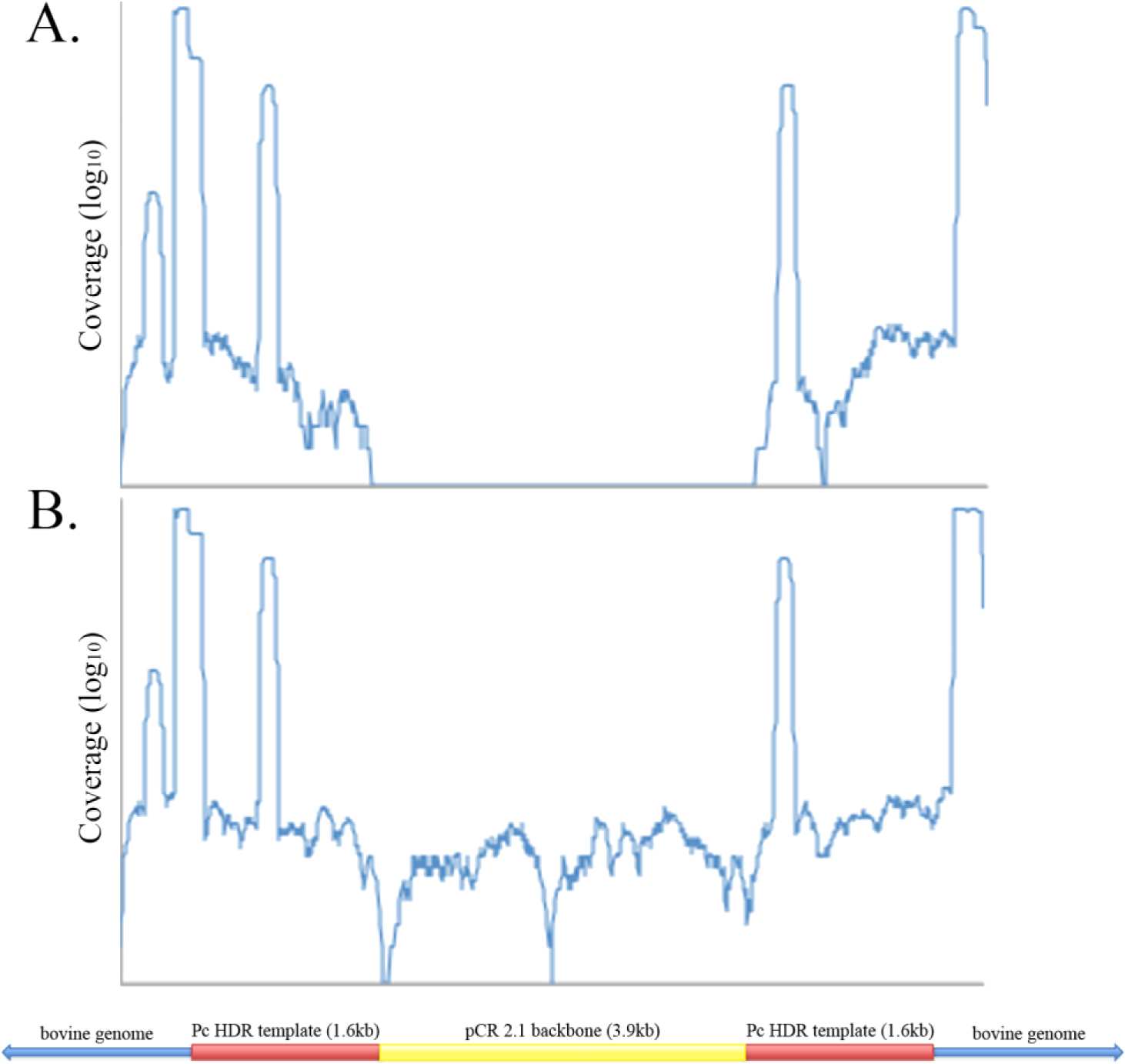

Supplement: Genomic sequence alignment to donor plasmid and PC homology-directed repair (HDR) template. — Alignment of the short-read genomic sequences to the pCR2.1 backbone (yellow) showed A) no read coverage across the backbone (n=23), or B) the presence of the plasmid in 5 of the animals (RCI002, RC.calf1, RC.calf4, RC.calf5, RC.calf6). [file 41587_2019_266_Fig11_ESM.jpg]
