# Supplementary material for: Genomic and phenotypic analyses of six offspring of a genome-edited hornless bull
Source: Nat Biotechnol. 2019 Oct 7;38(2):225–32. doi: 10.1038/s41587-019-0266-0 (PMC7007412; doi:10.1038/s41587-019-0266-0)
Supplement: Supplementary file 7 — Supplementary Figs. 1–6 and Tables 1–5. [file 41587_2019_266_MOESM1_ESM.pdf]

nature biotechnology

SUPPLEMENTARY INFORMATION

https://doi.org/10.1038/s41587-019-0266-0

In the format provided by the authors and unedited.

Genomic and phenotypic analyses of six offspring of a genome-edited hornless bull

Amy E. Young[1], Tamer A. Mansour[2,3], Bret R. McNabb[3], Joseph R. Owen[1], Josephine F. Trott[1], C. Titus Brown[3] and Alison L. Van Eenennaam[1*]

[1]Department of Animal Science, University of California, Davis, CA, USA. [2]Department of Clinical Pathology, School of Medicine, University of Mansoura, Mansoura, Egypt. [3]Department of Population Health and Reproduction, School of Veterinary Medicine, University of California, Davis, CA, USA.
*e-mail: alvaneenennaam@ucdavis.edu

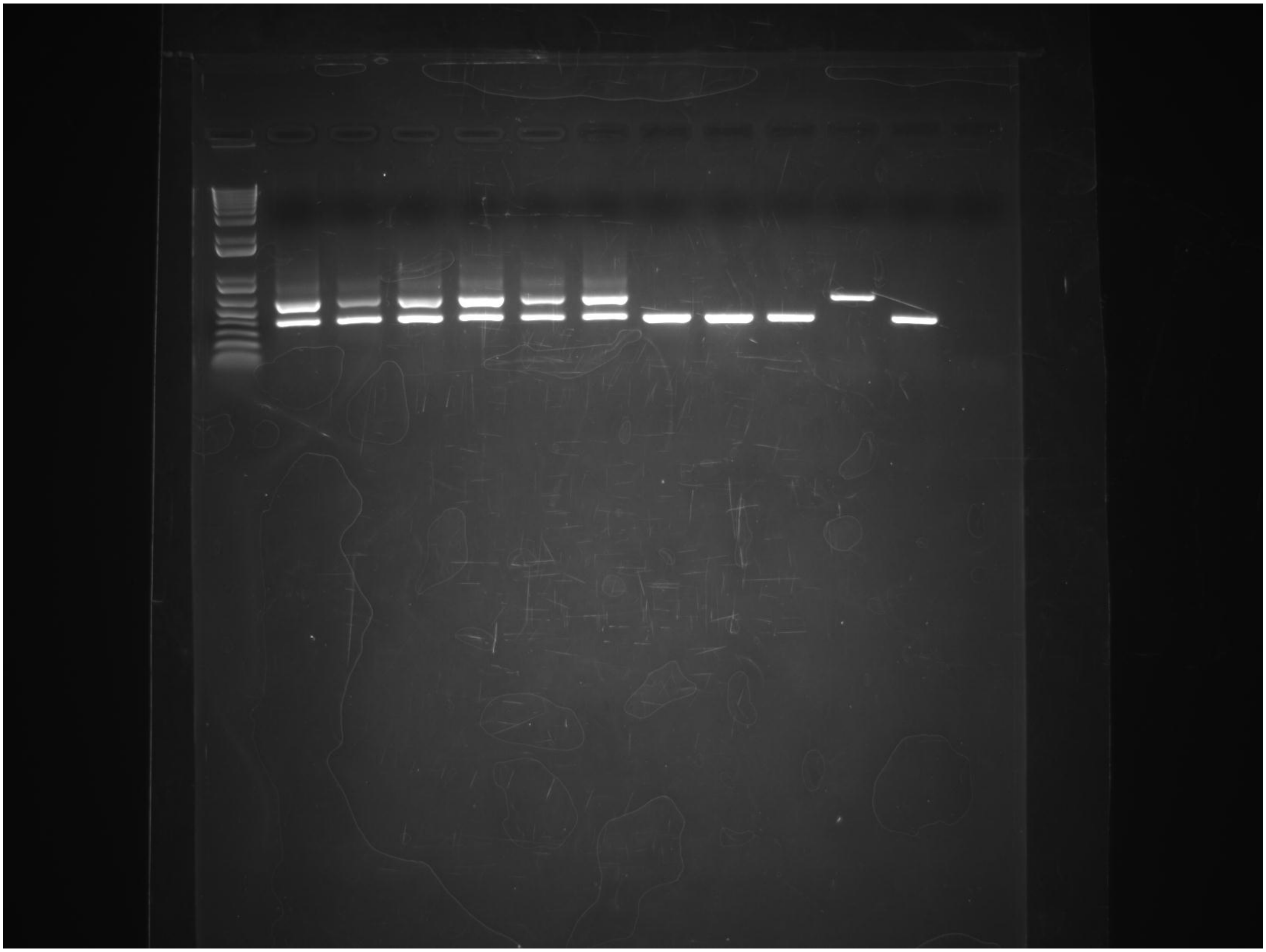

Supplementary Figure 1

Original scan of the PCR results depicted in Figure 3.

PCR products are shown for the genotyping of the polled locus for offspring of the genome-edited bull (first 6 lanes) and the Horned Hereford control offspring (next three lanes), with homozygous polled (P_CP_C; 591 bp), homozygous horned (pp; 389 bp), and negative PCR controls in the last 3 lanes. The molecular weight marker is in the far left lane.

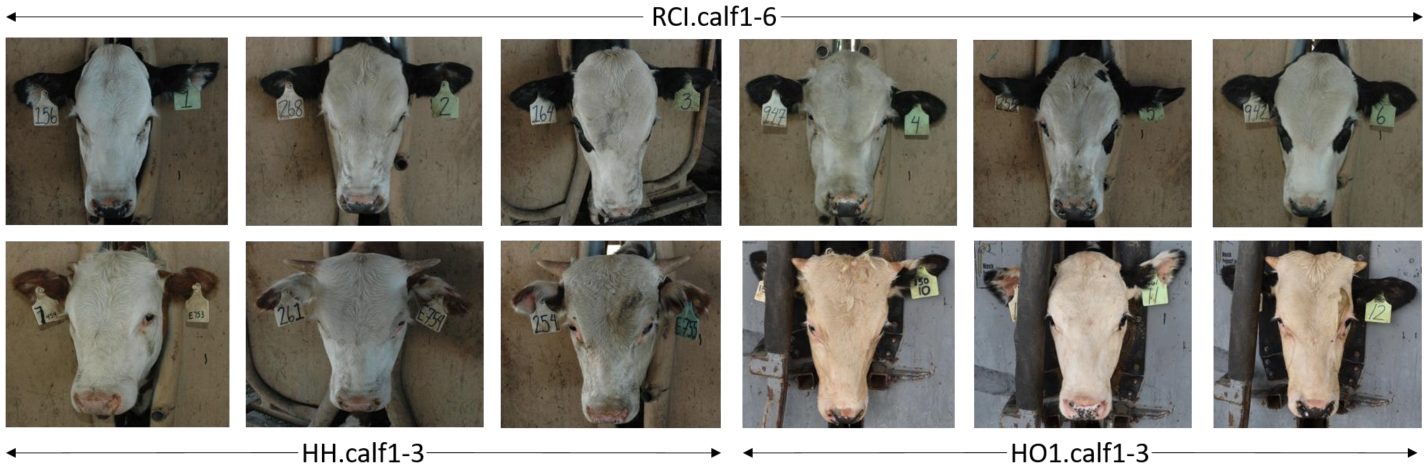

Supplementary Figure 2

Calves in this study at 8 months of age.

RCI.calf1-6 (top row) did not develop horns, whereas HH.calves1-3 and HO1.calves1-3 (bottom row) did develop horns.

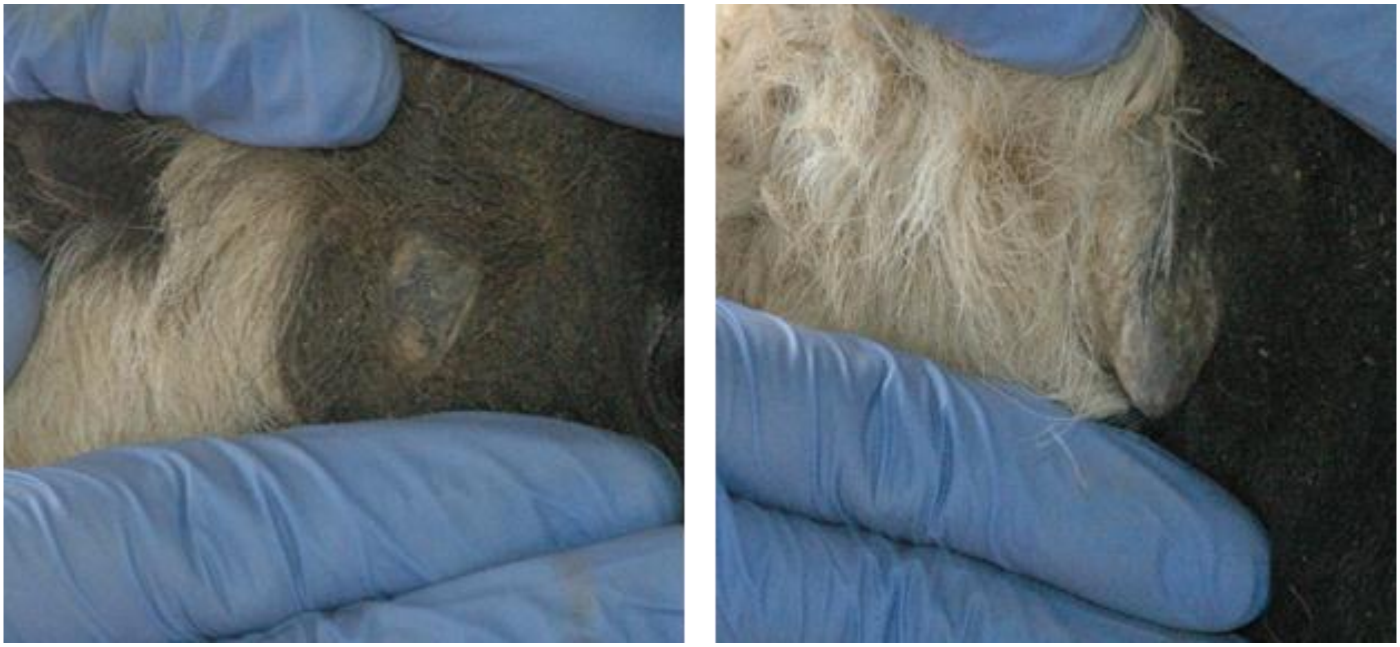

Supplementary Figure 3

Pictures of scurs on two of the five male offspring of the genome-edited bull.

All five heterozygous males showed scurs of varying sizes on one or both sides. Shown are bull calves RCI.calf5 (L) and RCI.calf6 (R).

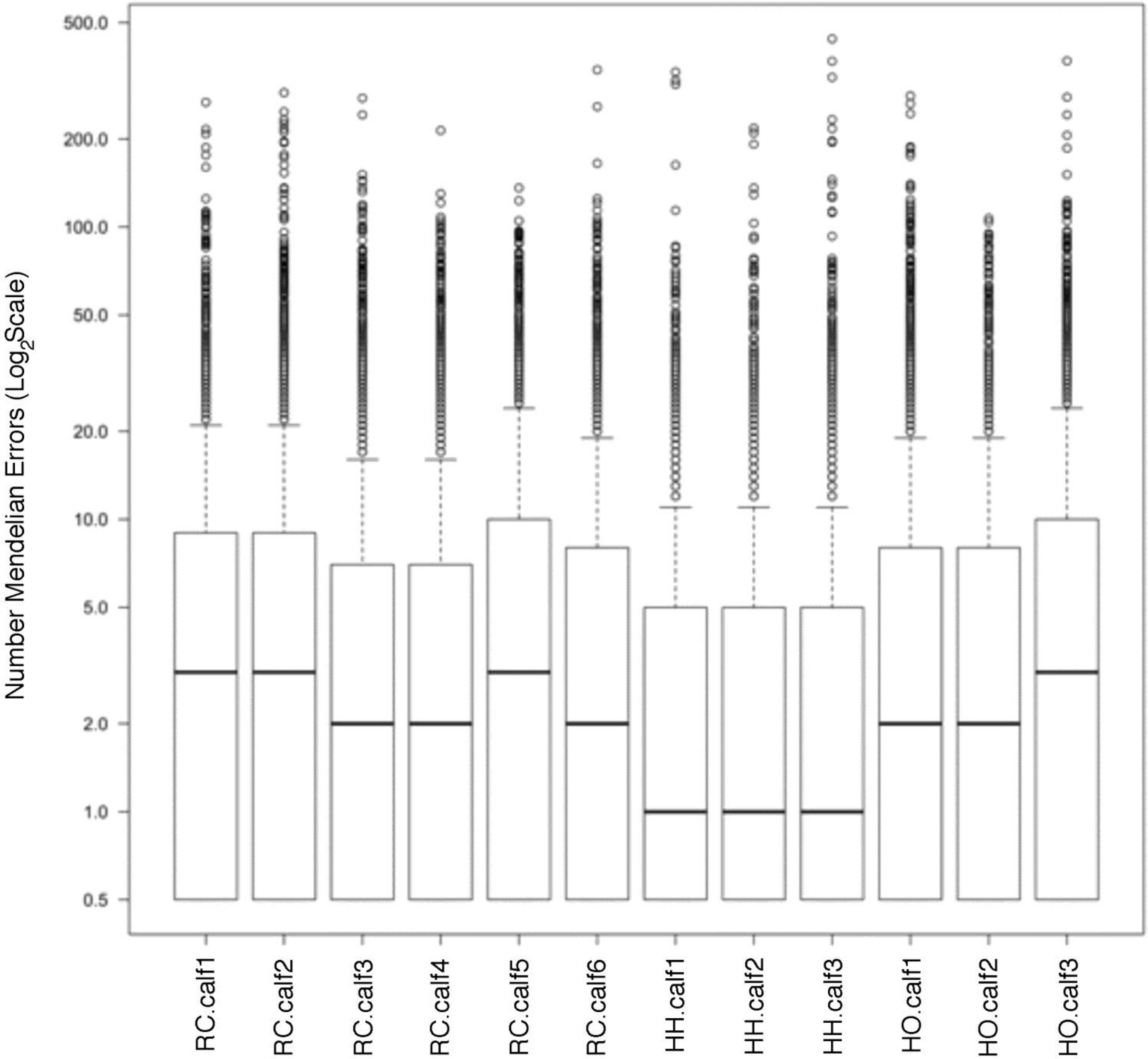

Supplementary Figure 4

Box plot of the number of Mendelian errors based the analysis of n = 12 biologically independent sire/dam/offspring trios at 4,438 10 kb regions of the genome with a high proportion of inherited errors.

Zero errors was set to 0.5 to allow for the log2 conversion. RC.calves1–6 are the offspring of the genome edited, polled bull. HH.calves1–3 are the Horned Hereford control offspring. HO1.calves1–3 are the control offspring from the Holstein bull. The figure was generated using the default parameters of the boxplot function in the package graphics version 3.5.1. Box-and-whisker plot: center line, median; bottom of box, 25% quartile (Q1); top of box, 75% quartile (Q3); whiskers, Q1 - 1.5 IQR and Q3 + 1.5 IQR where IQR is the interquartile range = Q3 - Q1.

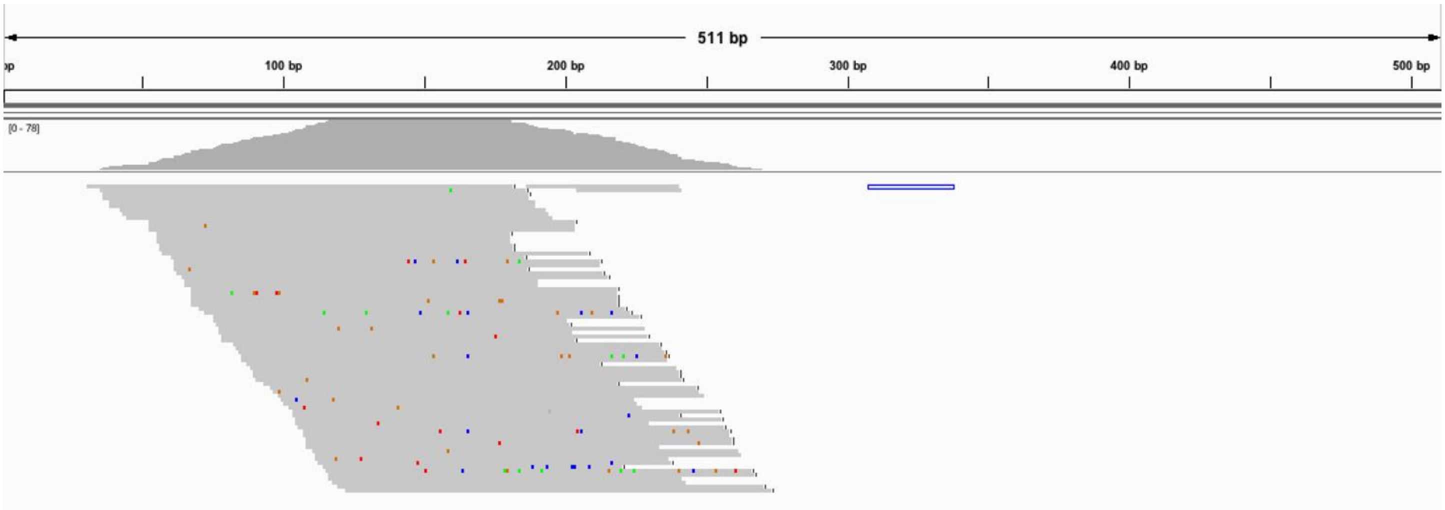

Supplementary Figure 5

Reads from heterozygous and homozygous P_C cattle shown mapping over the junction between the two repeats in the edited ARS-UCD1.2 bovine reference genome sequence that has the insertion sequence.

Note that although there are sequence variations indicated by colored dots, there is no consistent pattern suggesting sequencing errors rather than induced mutations. One read (colored in purple) mapped unexpectedly with supplementary alignment. Revised exact alignment of the read showed it should have mapped to the insertion position but had many sequencing errors that prevented the direct alignment to the expected locus.

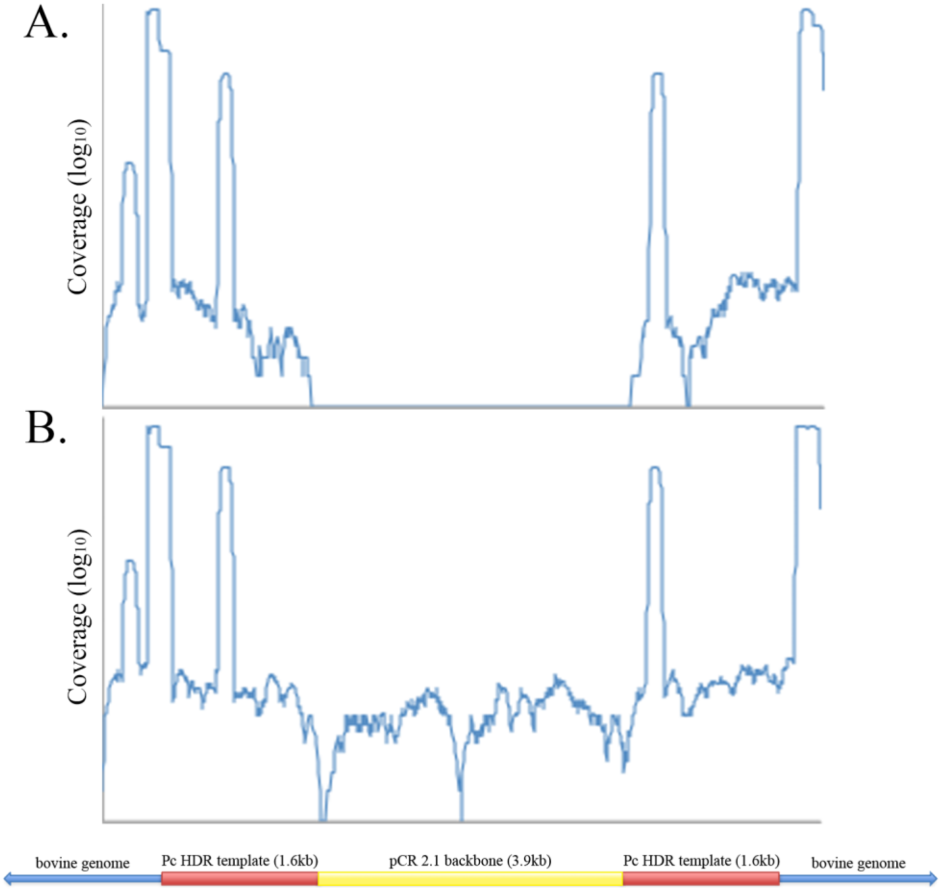

Supplementary Figure 6

Genomic sequence alignment to donor plasmid and P_C homology-directed repair (HDR) template.

Alignment of the short-read genomic sequences to the pCR2.1 backbone (yellow) showed A) no read coverage across the backbone (n=23), or B) the presence of the plasmid in 5 of the animals (RCI002, RC.calf1, RC.calf4, RC.calf5, RC.calf6).

Supplementary Table 1. The breakdown of heterozygous, compound heterozygous, and homozygous mutants for each animal as compared to the reference genome.

**Animal ID**	**SNPS**			**INDELs**		
	Homozygous mutant	Heterozygous	Compound Heterozygous	Homozygous mutant	Heterozygous	Compound Heterozygous
RCI002	2157950	4216164	8632	365155	604265	37652
RC.calf1	1531175	4319232	6507	279220	638834	34557
RC.calf2	1680899	4264309	6662	299906	628570	35125
RC.calf3	1683402	4294441	6810	296674	648579	39573
RC.calf4	1681629	4273913	7304	298620	637708	36807
RC.calf5	1617984	4187954	6579	294320	599438	29155
RC.calf6	1676353	4285226	7150	292082	662041	43138
RC.dam1	1472325	3353643	4527	280878	502373	22829
RC.dam3	1544344	3610549	5258	284600	560313	31609
RC.dam5	1492833	3449312	4872	281011	522736	26616
RC.dam2	1616242	3404615	4786	295451	524062	27161
RC.dam6	1584424	3454419	4830	292491	526962	26744
RC.dam4	1582340	3462411	5222	290277	535884	28026
HH.sire23	1512583	3251248	4552	288359	501225	27045
HH.sire1	1550511	3015825	4250	293640	447623	19979
HH.calf1	1563681	3227365	4571	290557	509157	27760
HH.calf2	1817861	2832508	4174	330149	445987	24168
HH.calf3	1505209	3380406	4990	281581	522333	26847
HH.dam3	1545544	3469588	5009	287997	522878	26055
HH.dam2	1596990	3365756	4378	297656	505738	24570
HH.dam1	1551655	3386972	4914	287520	528018	28751
HO1	2430750	3622040	7498	400510	536095	34920
HO1.calf1	1654391	4314997	7102	297972	635515	37069
HO1.calf2	1600163	4235890	6229	293914	609942	32465
HO1.calf3	1661594	4230967	6723	303301	609023	33200
HO1.dam1	1535270	3465764	4884	289560	524889	26579
HO1.dam2	1632251	3272158	4911	300734	514849	30390
HO1.dam3	1545873	3364389	4562	293745	492734	22996
RCI001-org	2090428	4189215	4139	339601	608652	26701
RCI002-org	2149642	4226214	4431	348652	615377	30405
Cellline2120-org	2087841	4205247	4261	338342	587440	23506
Cellline2122-org	2149707	4229252	4422	348343	623220	31579

Supplementary Table 2. Genomic 10kb intervals with high Mendelian error rate of more than 10 errors on average (i.e. > 1 error/kb) in all three (GH.H., H.H., and Ho.H) study groups.

Chromosome	Chromosome position of interval [(n-1)*10000 through (n*10000)-1].
3	857, 858
4	10571, 10599
5	402, 9921
8	2,3, 359
9	8709, 8710, 8715, 8724, 10436, 10437, 10439
10	2284, 2318, 2337, 2369, 2371, 2497, 2498, 2503, 10021, 10265
11	2654
12	4491, 7007, 7010, 7025, 7031, 7053, 7079, 7093, 7115, 7117, 7118, 7124, 7126, 7127, 7130, 7134, 7136, 7138, 7143, 7172, 7173, 7174, 7175, 7176, 7177, 7181, 7182, 7208, 7213, 7214, 7217, 7219, 7220, 7222,7223, 7224, 7226, 7227, 7228, 7229, 7230, 7231, 7232, 7233, 7234, 7235, 7236, 7237, 7238, 7240, 7241, 7242, 7243, 7244, 7245, 7246, 7258, 7259, 7260, 7261, 7262, 7263, 7264, 7265, 7266, 7268, 7269, 7270, 7271, 7276, 7279, 7280, 7281, 7282, 7283, 7284, 7285, 7286, 7288
16	590
17	7097, 7098, 7099, 7100, 7101, 7102
18	444, 6095, 6137, 6297, 6316
20	3825
22	1514
23	2558, 2559, 2565, 2566, 2567, 2568, 2569, 2570, 2571, 2595, 2596, 2600, 2601, 2605, 2606, 2607, 2608, 2609, 2610, 2611, 2612, 2613, 2614, 2615, 2616, 2628, 2629, 2630, 2641, 2642, 2643, 2644, 2645, 2646, 2647, 2648, 2649, 2650, 2685, 2864, 2866, 2867, 2870, 2873, 2874, 2902, 2904, 2920, 2927
28	2967
29	2734

Supplementary Table 3. GeneBank accession numbers for the 32 genomic sequences analyzed.

Seq	Sample_id	Sample_Title	Cluster_id	GeneBank SRA Accession	GeneBank Run Accession	Biosample Accession
30	RCI002	Genome edited Holstein (62.5% HO:25% MO:12.5% JE)	RCI002	SRX5136390	SRR8324591	SAMN10598549
		Long read whole genome PromethION sequence		SRX5900602	SRR9126620	N/A
2	1	Genome edited Holstein x Hereford (GH.H.) calf 1	RC.calf1	SRX5136385	SRR8324597	SAMN10598550
3	2	Genome edited Holstein x Hereford (GH.H.) calf 2	RC.calf2	SRX5136388	SRR8324593	SAMN10598551
4	3	Genome edited Holstein x Hereford (GH.H.) calf 3	RC.calf3	SRX5136387	SRR8324594	SAMN10598552
5	4	Genome edited Holstein x Hereford (GH.H.) calf 4	RC.calf4	SRX5136391	SRR8324590	SAMN10598553
6	5	Genome edited Holstein x Hereford (GH.H.) calf 5	RC.calf5	SRX5136389	SRR8324592	SAMN10598554
7	6	Genome edited Holstein x Hereford (GH.H.) calf 6	RC.calf6	SRX5136393	SRR8324588	SAMN10598555
8	156	Hereford dam for GH.H. calf 1	RC.dam1	SRX5136392	SRR8324589	SAMN10598556
29	164	Hereford dam for GH.H. calf 3	RC.dam3	SRX5136396	SRR8324585	SAMN10598557
31	255	Hereford dam for GH.H. calf 5	RC.dam5	SRX5136399	SRR8324582	SAMN10598558
11	268	Hereford dam for GH.H. calf 2	RC.dam2	SRX5136401	SRR8324580	SAMN10598559
12	942	Hereford dam for GH.H. calf 6	RC.dam6	SRX5136400	SRR8324581	SAMN10598560
13	947	Hereford dam for GH.H. calf 4	RC.dam4	SRX5136403	SRR8324578	SAMN10598561
21	HH2381	Hereford sire for H.H. calfs 2&3	HH.sire23	SRX5136410	SRR8324572	SAMN10598569
22	HH2981	Hereford sire for H.H. calf 1	HH.sire1	SRX5136408	SRR8324573	SAMN10598570
14	E753	Hereford x Hereford (H.H.) calf 1	HH.calf1	SRX5136402	SRR8324579	SAMN10598562
15	E754	Hereford x Hereford (H.H.) calf 2	HH.calf2	SRX5136405	SRR8324576	SAMN10598563
16	E755	Hereford x Hereford (H.H.) calf 3	HH.calf3	SRX5136404	SRR8324577	SAMN10598564
17	254	Hereford dam for H.H. calf 3	HH.dam3	SRX5136407	SRR8324574	SAMN10598565
18	261	Hereford dam for H.H. calf 2	HH.dam2	SRX5136406	SRR8324575	SAMN10598566
19	459	Hereford dam for H.H. calf 1	HH.dam1	SRX5136398	SRR8324583	SAMN10598567
20	HO1	Holstein Sire for Ho.H. calfs 1&2&3	HO1	SRX5136395	SRR8324586	SAMN10598568
23	10	Holstein x Hereford (Ho.H.) calf 1	HO1.calf1	SRX5136411	SRR8324571	SAMN10598571
24	11	Holstein x Hereford (Ho.H.) calf 2	HO1.calf2	SRX5136412	SRR8324569	SAMN10598572
27	12	Holstein x Hereford (Ho.H.) calf 3	HO1.calf3	SRX5136414	SRR8324567	SAMN10598575
25	150	Hereford dam for Ho.H. calf 1	HO1.dam1	SRX5136409	SRR8324570	SAMN10598573
26	161	Hereford dam for Ho.H. calf 2	HO1.dam2	SRX5136413	SRR8324568	SAMN10598574
28	50	Hereford dam for Ho.H. calf 3	HO1.dam3	SRX5136415	SRR8324595	SAMN10598576
1	RCI001	WGS of RCI_001	RCI001-org	SRX1660368	SRR3290535	SAMN04576207
2	RCI002	WGS of RCI_002	RCI002-org	SRX1660380	SRR3290615	SAMN04576211
3	Cellline2120	Holstein Cell line	Cellline2120-org	SRX1660381	SRR3290631	SAMN04576212
4	Cellline2122	Holstein Cell line	Cellline2122-org	SRX1660382	SRR3290632	SAMN04576214

Supplementary Table 4. PCR primers used to amplify the pCR2.1 plasmid backbone from genomic DNA.

Primer	Sequence (5’ – 3’)	Annealing Temperature (°C)
topoIF	GCCTTCTCCGAGGGACATA	62.3
topoIR	CAGTTGTGGGGAAGGAAAAA	58.4
M13F	GTAAAACGACGGCCAGT	57.2
M13R	CAGGAAACAGCTATGAC	54.8

Supplementary Table 5. Primers and probes for qPCR.

Trait	Primers	Sequence (5’ – 3’)	Probe	Sequence (5’-3’)
Polled	btPolled-213f	GCAGGCTGGAATTATGTCTGG	btPolled-250p	FAM-TGGTAGGCTGTGAAATG-MGB
	btPolled-312r	TCCAGCTGTGCGTGAGCTC		
Horned	btHorned-213f	GCAGGCTGGAATTATGTCTGG	btHorned-254p	FAM-AGGCTGGTATTCTTG-MGB
	btHorned-333r	TCAAGGACACCTCCCACACTT		
Y chromosome	btYchr-26f	GGACTCCACGCTTTCCTATCAG	btYchr-60p	FAM-GGTTCTCAACCAGGAAA-MGB
	btYchr-102r	GCTGTGCTATGCAGCTTGAATG		
HBB – housekeeping gene	btHBB-1677f	TGCAGGCTGACTTTCAGAAGG	btHBB-1701p	FAM-TGGCTGGTGTGGCCA-MGB
	btHBB-1744r	AGCTTAATGATATCTGTGGGCCA		
