# Supplementary material for: Genomic and phenotypic analyses of six offspring of a genome-edited hornless bull
Source: Nat Biotechnol. 2019 Oct 7;38(2):225–32. doi: 10.1038/s41587-019-0266-0 (PMC7007412; doi:10.1038/s41587-019-0266-0)
Supplement: Supplementary file 8 — Reporting Summary [file 41587_2019_266_MOESM2_ESM.pdf]

nature research

Corresponding author(s): A. L. Van Eenennaam

Last updated by author(s): Aug 16, 2019

Reporting Summary

Nature Research wishes to improve the reproducibility of the work that we publish. This form provides structure for consistency and transparency in reporting. For further information on Nature Research policies, see Authors & Referees and the Editorial Policy Checklist.

Statistics

For all statistical analyses, confirm that the following items are present in the figure legend, table legend, main text, or Methods section.

n/a	Confirmed	
☐	☒	The exact sample size (n) for each experimental group/condition, given as a discrete number and unit of measurement
☐	☒	A statement on whether measurements were taken from distinct samples or whether the same sample was measured repeatedly
☐	☒	The statistical test(s) used AND whether they are one- or two-sided<br>*Only common tests should be described solely by name; describe more complex techniques in the Methods section.*
☒	☐	A description of all covariates tested
☒	☐	A description of any assumptions or corrections, such as tests of normality and adjustment for multiple comparisons
☐	☒	A full description of the statistical parameters including central tendency (e.g. means) or other basic estimates (e.g. regression coefficient) AND variation (e.g. standard deviation) or associated estimates of uncertainty (e.g. confidence intervals)
☐	☒	For null hypothesis testing, the test statistic (e.g. F, t, r) with confidence intervals, effect sizes, degrees of freedom and P value noted<br>*Give P values as exact values whenever suitable.*
☒	☐	For Bayesian analysis, information on the choice of priors and Markov chain Monte Carlo settings
☐	☒	For hierarchical and complex designs, identification of the appropriate level for tests and full reporting of outcomes
☒	☐	Estimates of effect sizes (e.g. Cohen's d, Pearson's r), indicating how they were calculated

Our web collection on statistics for biologists contains articles on many of the points above.

Software and code

Policy information about availability of computer code

Data collection	MinKnow v19.01.6, PLINK 1.9, 'ape' package in R, BWA v0.7.17, FastQC v0.11.7, multiqc v1.0, Trimmomatic v 0.36, BWA-MEM (BWA v0.7.7), Picard v2.18.1, GATK v4.0.90, Primer3
Data analysis	The code used in this study is available on GitHub at https://github.com/dib-lab/Bovine_seq.

For manuscripts utilizing custom algorithms or software that are central to the research but not yet described in published literature, software must be made available to editors/reviewers. We strongly encourage code deposition in a community repository (e.g. GitHub). See the Nature Research guidelines for submitting code & software for further information.

Data

Policy information about availability of data

All manuscripts must include a data availability statement. This statement should provide the following information, where applicable:

- Accession codes, unique identifiers, or web links for publicly available datasets
- A list of figures that have associated raw data
- A description of any restrictions on data availability

Whole genome sequences have been deposited in the NBCI Sequence Read Archive under BioProject PRJNA494431. Sequences from Carlson et al., 2016 are under BioProject PRJNA3161222. See Supplementary Table 2 for a full list of accession numbers. Figures 3, 4 and 5 are based on the raw data contained in the sequence data. There are no restrictions on data availability.

Field-specific reporting

Please select the one below that is the best fit for your research. If you are not sure, read the appropriate sections before making your selection.

☒ Life sciences ☐ Behavioural & social sciences ☐ Ecological, evolutionary & environmental sciences

For a reference copy of the document with all sections, see nature.com/documents/nr-reporting-summary-flat.pdf

Life sciences study design

All studies must disclose on these points even when the disclosure is negative.

Sample size	Initially we inseminated 10 cows with the goal of obtaining at least one offspring of each gender. No statistical methods were used to predetermine sample sizes. Studies have shown pregnancy rates after artificial insemination to be approximately 60%. We used this information, the practical availability of cows at our facility, and the binary nature of the expected phenotype (+/- horns) to determine the number that we bred. We actually obtained 6 offspring, 5 males and one female.
Data exclusions	No data was excluded from the analysis
Replication	Animals were evaluated at birth, 4 months and one year of age to confirm horned/polled phenotype. The phenotype was replicated at every age tested.
Randomization	The experimental animals were the offspring of the gene edited bull. Control animals were horned animals sired by the sire of the gene-edited bull or Horned Hereford bulls.
Blinding	The experimental animals that were the offspring of the gene edited bull were phenotypically apparent as they had no horns, as compared to the controls. The laboratory personnel that ran the blood work and the TaqMan PCR for fetal microchimerism were blind as to which sample belonged to which group.

Reporting for specific materials, systems and methods

We require information from authors about some types of materials, experimental systems and methods used in many studies. Here, indicate whether each material, system or method listed is relevant to your study. If you are not sure if a list item applies to your research, read the appropriate section before selecting a response.

Materials & experimental systems

n/a	Involved in the study	
☒	☐	Antibodies
☒	☐	Eukaryotic cell lines
☒	☐	Palaeontology
☐	☒	Animals and other organisms
☒	☐	Human research participants
☒	☐	Clinical data

Methods

n/a	Involved in the study	
☒	☐	ChIP-seq
☒	☐	Flow cytometry
☒	☐	MRI-based neuroimaging

Animals and other organisms

Policy information about studies involving animals; ARRIVE guidelines recommended for reporting animal research

Laboratory animals	Bos Taurus, 28 animals of different breeds and genders, from newborn to adult
Wild animals	The study did not involve wild animals.
Field-collected samples	The study did not involve samples collected from the field.
Ethics oversight	UC Davis Institutional Animal Care and Use Committee (protocol #18855)

Note that full information on the approval of the study protocol must also be provided in the manuscript.
